# Therapeutic Mesenchymal Stem/Stromal Cells: Value, Challenges and Optimization

**DOI:** 10.3389/fcell.2021.716853

**Published:** 2022-01-14

**Authors:** Mehdi Najar, Rahma Melki, Ferial Khalife, Laurence Lagneaux, Fatima Bouhtit, Douaa Moussa Agha, Hassan Fahmi, Philippe Lewalle, Mohammad Fayyad-Kazan, Makram Merimi

**Affiliations:** ^1^ Laboratory of Clinical Cell Therapy, Institut Jules Bordet, Université Libre de Bruxelles (ULB), Brussels, Belgium; ^2^ Osteoarthritis Research Unit, University of Montreal Hospital Research Center (CRCHUM), Montreal, QC, Canada; ^3^ Genetics and Immune-Cell Therapy Unit, LBBES Laboratory, Faculty of Sciences, University Mohammed Premier, Oujda, Morocco; ^4^ Laboratory of Cancer Biology and Molecular Immunology, Faculty of Sciences I, Hadath, Lebanon; ^5^ Laboratory of Experimental Hematology, Institut Jules Bordet, Université Libre de Bruxelles (ULB), Bruxelles, Belgium; ^6^ Department of Natural Sciences, School of Arts and Sciences, Lebanese American University, Hadath, Lebanon; ^7^ Laboratory of Cancer Biology and Molecular Immunology, Faculty of Sciences-I, Lebanese University, Hadath, Lebanon

**Keywords:** mesenchymal stem/stromal cells, therapeutic features, clinical value, challenges, optimization

## Abstract

Cellular therapy aims to replace damaged resident cells by restoring cellular and molecular environments suitable for tissue repair and regeneration. Among several candidates, mesenchymal stem/stromal cells (MSCs) represent a critical component of stromal niches known to be involved in tissue homeostasis. *In vitro*, MSCs appear as fibroblast-like plastic adherent cells regardless of the tissue source. The therapeutic value of MSCs is being explored in several conditions, including immunological, inflammatory and degenerative diseases, as well as cancer. An improved understanding of their origin and function would facilitate their clinical use. The stemness of MSCs is still debated and requires further study. Several terms have been used to designate MSCs, although consensual nomenclature has yet to be determined. The presence of distinct markers may facilitate the identification and isolation of specific subpopulations of MSCs. Regarding their therapeutic properties, the mechanisms underlying their immune and trophic effects imply the secretion of various mediators rather than direct cellular contact. These mediators can be packaged in extracellular vesicles, thus paving the way to exploit therapeutic cell-free products derived from MSCs. Of importance, the function of MSCs and their secretome are significantly sensitive to their environment. Several features, such as culture conditions, delivery method, therapeutic dose and the immunobiology of MSCs, may influence their clinical outcomes. In this review, we will summarize recent findings related to MSC properties. We will also discuss the main preclinical and clinical challenges that may influence the therapeutic value of MSCs and discuss some optimization strategies.

## 1 Introduction

Mesenchymal stem/stromal cells (MSCs) and their secretome have been investigated for the treatment of several medical indications. Establishing a clear definition and characterization of MSCs (including their origin, terminology and identity), identifying the major preclinical and clinical challenges linked to their application and finally proposing suitable therapeutic optimization strategies may help highlight the value of MSCs and therefore develop stem cell-based therapy.

**GRAPHICAL ABSTRACT:**
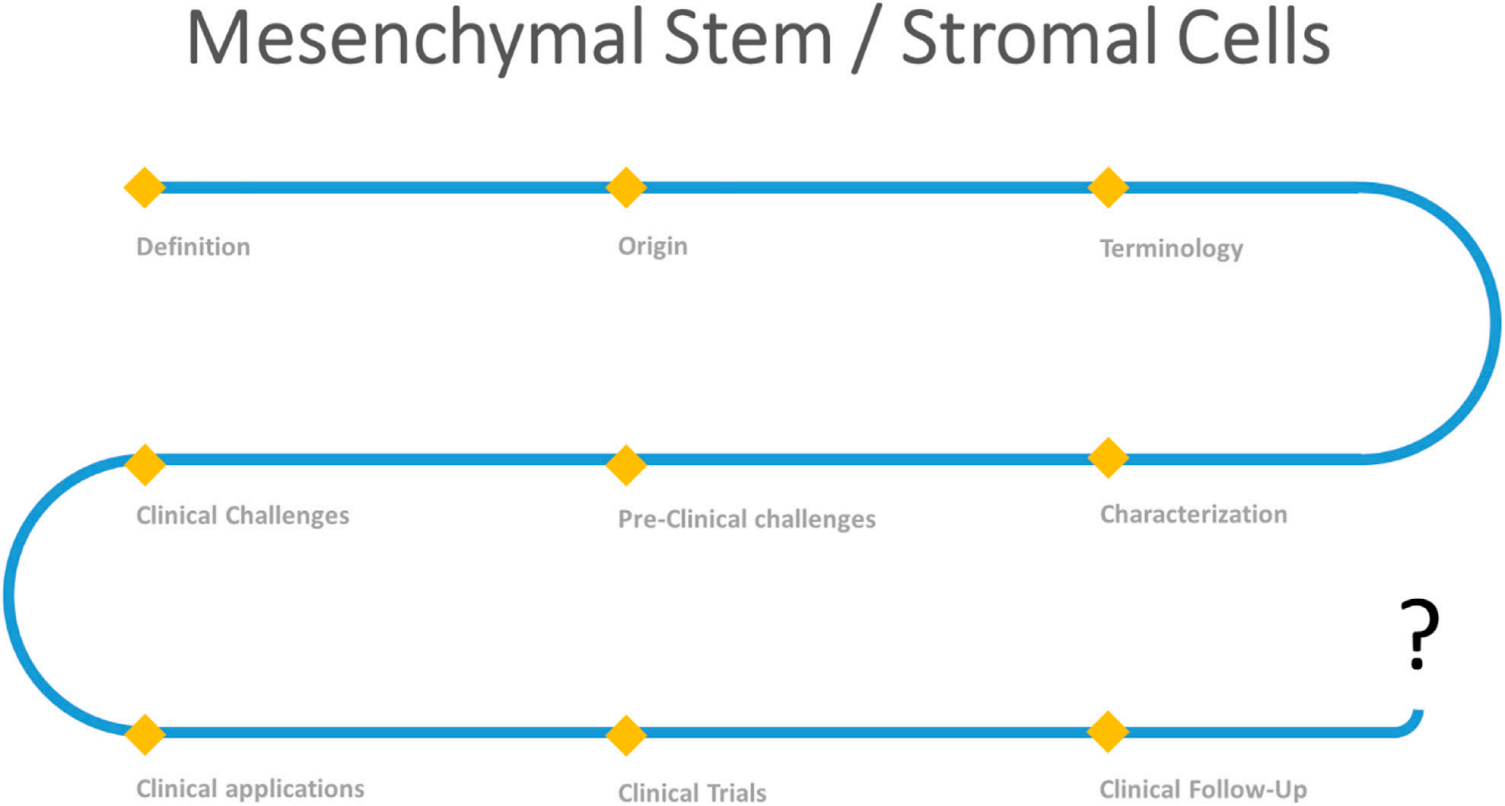
The road map for the use of MSCs.

### 1.1 Definition and Origin

MSCs represent a heterologous subset of nonhematopoietic precursors that are broadly distributed throughout the body. The ontogenic origin of MSCs is still controversial. In addition to their most widely accepted mesodermal origin, MSCs may originate in part from neuroepithelial tissue or perivascular tissue (Huang et al., 2011). Currently, it has been established that MSCs can be isolated from all vascularized tissues since MSCs reside in the walls of blood vessels, forming part of the endothelium (Caplan, 2009). MSCs can be derived from a variety of tissues, including the bone marrow, adipose tissue, peripheral blood, umbilical cords, Wharton’s jelly, dental pulp and other tissues (Kumar et al., 2019). MSCs have been investigated in the field of cellular therapy and regenerative medicine to treat a variety of diseases and disorders (Kabat et al., 2020). This research interest is due to the relatively easy and minimally invasive access to MSCs as well as their several properties. MSCs harbor immunomodulatory, anti-inflammatory, angiogenic, antioxidative and antiapoptotic capacities (Wei et al., 2013; Kaukua et al., 2014). As will be discussed, these effects mainly involve paracrine pathways rather than direct cell differentiation (Wu et al., 2020; Xunian and Kalluri, 2020). MSCs present a high self-renewing capacity that enables their *ex vivo* expansion to obtain a sufficient number of cells for clinical purposes (Berebichez-Fridman and Montero-Olvera, 2018). Because MSCs generate most of the stromal cells present in the bone marrow (BM), form part of the hematopoietic stem cell (Klein et al., 2018) niche, and produce various molecules regulating hematopoiesis, their hematopoiesis-supporting capacity has been demonstrated (Fajardo-Orduna et al., 2015). These features allow the use of MSCs for the *in vitro* expansion of HSCs before their transplantation (Sensebe et al., 1995; Yamaguchi et al., 2001).

### 1.2 Terminology

**FIGURE 1:**
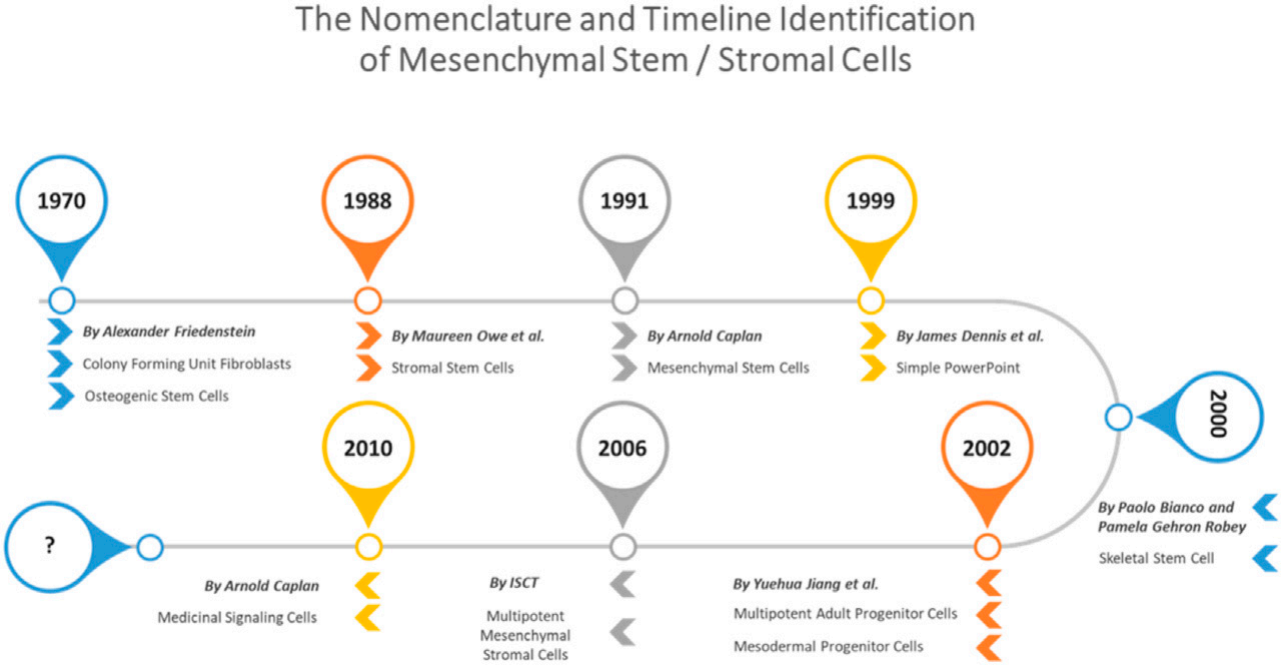
The nomenclature of MSCs over the time.

The terminology of MSCs has considerably evolved over time (Figure 1). Since their initial characterization, these cells were referred to as MSCs, an acronym that was used to indicate “Mesenchymal Stromal Cells,” “Mesenchymal Stem Cells,” “Multipotent Stromal Cells,” “Mesodermal Stem Cells” (Caplan, 2017), “skeletal stem/progenitor cells” (Bianco, 2011), “mesenchymal progenitor cells” and “pericytes mesenchymal stem cells” (Caplan, 2017). Initially, Friedenstein termed those cells “mechanocytes” or osteogenic stem cells” and then “marrow stromal cells”. In subsequent work, those cells were designated “marrow fibroblasts” (Friedenstein et al., 1970). In the 1980s, the term “marrow stromal cells” was adopted to distinguish mesenchymal stromal cells that were able to maintain hematopoiesis for many weeks *in vitro* from other adherent hematopoietic cells, such as macrophages and marrow fibroblasts (Keating et al., 1984). In 1991, Caplan proposed the term “mesenchymal stem cells” due to their multilineage differentiation potential. Although the term became popular and was widely used, the mesenchymal stem cell nomenclature proved to be problematic when it became obvious that not all plastic adherent stromal cells have comparable self-renewal and *in vivo* differentiation ability into multiple lineages. More recently, Caplan recommended designating these cells “medicinal signaling cells” to highlight the mechanism underlying their therapeutic effects after transplantation, which is believed to be based mainly on the secretion of a plethora of anti-inflammatory, antiapoptotic, proangiogenic and immunosuppressive factors facilitating regenerative processes (Caplan, 2017).

Given that MSCs are now being derived from different tissue sources and exhibit distinct phenotypes and functions, a consortium of international MSC investigators held a series of workshops to address the challenges facing the field, including a reassessment of MSC nomenclature. The consensus of the Nomenclature Working Group has recommended the following terminology for mesenchymal stromal cells: donor type (autologous or allogeneic), species of origin (e.g., human, mouse), tissue source (e.g., bone marrow, adipose tissue, cord blood), and mesenchymal cell type (stem cell, stromal cell or progenitor cell) (Bourin et al., 2013). As proposed by Bhartiya D et al., it is better to revisit the definition of MSCs based on their functional attributes (Bhartiya, 2018).

### 1.3 Characterization of Mesenchymal Stem/Stromal Cells

Many research groups have used distinct tissue sources and developed different protocols for MSC isolation, cultivation and expansion, which have resulted in heterogeneous populations of cells and difficulties in comparing experimental outcomes. This difficulty is, in part, due to the lack of a definitive marker of MSCs (Viswanathan et al., 2019).

Due to the growing controversy regarding the nomenclature, degree of stemness and characteristics of MSCs, the International Society for Cellular Therapy (ISCT) published two important reports to address these limitations. The first report clarified the terminology, emphasizing that while most mesenchymal stem or stromal cells are not stem cells, the bulk population represents a multipotent mesenchymal stromal cell population (Horwitz et al., 2005). In the second report, ISCT recommended the usage of “multipotent mesenchymal stromal cells” (MSCs) to refer to the plastic-adherent fraction of stromal tissues regardless of their origin capable of *in vitro* differentiation into specific, multiple cell lineages (Dominici et al., 2006).

To standardize the isolation and characterization of MSCs *in vitro*, ISCT has specified the minimum inclusion criteria for defining MSCs. These criteria include:

1) The ability to adhere to plastic under standard culture conditions;
2) The phenotypic expression of surface markers such as CD73, CD105 and CD90. The absence of a series of surface markers, including CD14, CD19, CD34, and CD45.
3) The ability to differentiate into osteoblasts, chondrocytes and adipocytes *in vitro* under the effect of specific culture media (Dominici et al., 2006).

Later, ISCT proposed criteria for the immunological characterization of MSCs. These include 1) MSC response to interferon-γ (IFN-γ) and tumor necrosis factor-α (TNF-α); 2) indoleamine 2,3-dioxygenase (IDO) response in cytokine licensing assays; 3) assessment of the functionality of the expanded cell product; 4) usage of purified immune responders in functional assays; 5) analysis of mechanistic and efficacy studies of human MSCs in xenotransplantation models; 6) immune reaction to infused MSCs; and 7) analysis of the lymphocyte populations of patients treated with MSCs (Krampera et al., 2013).

### 1.4 Therapeutical Potential of Mesenchymal Stem/Stromal Cells

Over the past decades, a large number of studies have emerged using MSC-based therapies in preclinical studies to treat many different pathologies, including cardiovascular diseases, bone and cartilage diseases and immune-/inflammation-mediated diseases (Damasceno et al., 2020). However, the therapeutic potential of MSCs in cancer treatment is still controversial. Depending on several parameters, MSCs have been shown to either promote or suppress tumor development (Hmadcha et al., 2020). Because of this reality, a sustained effort to understand such duality before planning an MSC-based therapy for cancer is required. Herein, we provide an overview of some preclinical and clinical studies that may highlight the value of MSCs.

#### 1.4.1 Preclinical Models

MSCs represent a primary choice for treating immunological disorders such as acute graft-versus-host disease (GvHD) and Crohn’s disease (Galipeau and Sensebe, 2018; Carvello et al., 2019). A meta-analysis of 50 studies involving 1,848 animals showed that MSCs significantly prevented mortality and alleviated the clinical manifestations of GvHD (Wang et al., 2019). MSCs represent optimistic hope in treating rheumatoid arthritis (RA), as there is currently no effective treatment for this chronic inflammatory autoimmune disorder (Liu et al., 2020). Intravenous injections of MSCs derived from the human umbilical cord in a mouse model of RA showed a promising therapeutic effect (Yu et al., 2019). A preclinical study using MSCs derived from human adipose tissue reported that several mechanisms were involved in the therapeutic benefit of MSCs for treating RA (Zhou et al., 2011). Using BM-MSCs with induced colony-stimulating factor-1 in a mouse CCL4 (C-C Motif Chemokine Ligand 4) model of cirrhosis synergistically improved reduced liver fibrosis and improved hepatocyte proliferation (Watanabe et al., 2019).

A study performed on a guinea pig model using human bone marrow-derived MSCs (BM-MSCs) combined with hydroxyapatite scaffolds to treat temporal bone defects showed promising results, as the treatment was safe and effective and improved the repair of bone defects (Skoloudik et al., 2018). Another study using human umbilical cord-derived MSCs showed optimistic results in treating vertebral bone defects in weaned rabbits (Cui et al., 2019). Mouse BM-MSCs were shown to attenuate ischemia-reperfusion brain injury and inhibit microglial apoptosis (Cheng et al., 2021). Human umbilical cord MSCs (UC-MSCs) effectively improved renal function and inhibited inflammation and fibrosis in a rat model of diabetic nephropathy (DN) (Xiang et al., 2020). BM-MSCs have been shown to be protective in a rat model of renal ischemia reperfusion injury (Quirici et al., 2002) by inhibiting cell apoptosis and inflammatory responses (Li et al., 2019).

As MSCs are regulators of tissue homeostasis, they are also a promising material for the restoration of skeletal muscles after injury. Although skeletal muscle recovery is mainly provided by muscle stem cells, namely, satellite cells (MuSCs), MSCs may also participate in such regeneration. Thus, MSCs appear to be a promising approach for the restoration of skeletal muscle structure and function. Different studies have reported the positive effects of MSCs on the repair and regeneration of injured skeletal muscle tissue. It was observed that rat BM-MSCs do not show potency for myogenic differentiation *in vitro* under the influence of appropriate inducers and rarely fuse with myoblasts when cocultured. However, the BM-MSCs stimulate the differentiation of muscle tissue cells through paracrine secretion (Sheveleva et al., 2020). As reviewed by Qazi et al. (2019) MSCs from different sources have been shown to improve muscle contractility and structure and reduce inflammation in various muscle injury models. Both BM-MSCs and AT-MSCs improved the regeneration of skeletal muscle laceration injury at short- and long-term durations (rat models). Effective reinnervation of injured muscles occurred only in the long term. However, AT-MSCs showed better regenerative effects, evidenced by a significant increase in the number of myotubes and a significant decrease in collagen deposition (Moussa et al., 2020). MSCs can promote angiogenesis, cell recruitment, migration, proliferation and differentiation within the site of injury. They can also modulate the immune cell population surrounding the injured muscle (Qazi et al., 2019). In particular, MSCs are able to induce the proliferation and differentiation of resident MuSCs and are also able to act on other cellular components of the muscle cell niche by reducing inflammation and infiltration (Sandona et al., 2021). Several studies have demonstrated that the efficacy of MSCs in supporting skeletal muscle regeneration is linked to their secretome. The production of many biologically active factors with a wide spectrum of action may explain the effects of MSCs. Within the site of damage, these factors can exert a bioactive effect either by acting directly on muscle cell populations or by modulating the local environment. As indicated by Wang et al., several signaling pathways may participate in skeletal muscle regeneration (Wang et al., 2020). Using MALDI imaging mass spectrometry, a study revealed the early molecular processes of muscle healing upon treatment with MSCs and highlighted the critical role of trauma-adjacent tissue during the therapeutic response (Klein et al., 2018). Proteomic profiling highlighted that enriched pathways related to extracellular matrix organization, axon guidance, antigen processing, metabolic processes, immunomodulation and positive regulation of nitric oxide are also involved in muscle regeneration by MSCs (Sandona et al., 2021). *In vitro*, *in vivo* and bioinformatic results showed that MSCs promote skeletal muscle regeneration through the synergistic action of EVs and the soluble fraction of the secretome (Mitchell et al., 2019). Thus, several regulators of muscle regeneration, such as ectodysplasin-A2, thrombospondin-1, IL-6, monocyte chemoattractant protein-1 (MCP-1), dickkopf-related protein 1 (DKK1), HGF, VEGF, FGF7, tissue inhibitor of metalloproteinase 1 (TIMP-1), SMAD family member 4 (SMAD4), macrophage inflammatory protein 2 (MIP-2), activin A, insulin-like growth factor-binding protein (IGFBP)-related protein 1 and MMP-1, have been identified (Kim et al., 2016). Recently, a study provided compelling experimental evidence of the ability of the MSC secretome to exert a protective effect against eccentric contraction (EC)-induced skeletal myofiber damage (murine model). The secretome was able to modulate the behavior of SCs, which are key players in muscle tissue regeneration (Squecco et al., 2021). The long-term effectiveness of BM-MSCs for skeletal muscle regeneration was clearly established after 1 year of treatment. Using a pig model of severe radiation burn, local injection of BM-MSCs improved recovery skeletal muscle damage by acting on muscle regenerative capacity, muscle fibrosis and angiogenesis (Linard et al., 2018). Moreover, combination therapy between MSCs and muscle progenitor cells also enhanced skeletal muscle regeneration in muscular dystrophies. In this case, BM-MSCs create an appropriate muscle pro-regenerative environment by secreting trophic factors that regulate the proliferation and differentiation of muscle progenitor cells as well as immunomodulatory factors to manage local inflammation (Klimczak et al., 2018). EVs produced by MSCs of different tissue origins can influence myogenesis and fibrosis, the main processes that accompany skeletal muscle regeneration. Novokreshchenova et al. (2020) found that EVs derived from rat MSCs of distinct sources (BM, AT, intact muscle) significantly increased the number of newly formed myotubes in myoblast culture *in vitro* and reduced the number and size of fibrotic nodules in muscle fibroblast culture *in vitro*. In a previous report, exosomes from BM-MSCs were shown to promote myogenesis and angiogenesis *in vitro* and muscle regeneration in a mouse model of cardiotoxin-induced muscle injury. Although these exosomes had low concentrations of muscle repair-related cytokines, a number of repair-related miRNAs were identified (Nakamura et al., 2015). A system of MSC-encapsulated fibrin microbeads demonstrated effectiveness in shortening the regeneration period of volumetric muscle loss injury in a rat model (Lalegul-Ulker et al., 2019). We see forward to future studies developing new strategies allowing a full characterization of the profile of the factors contained in the MSC secretome and, therefore, a clear identification of the mechanisms underpinning its protective action.

Stem cell therapies are among the most promising regenerative approaches for cardiovascular diseases. Several animal studies have shown that MSCs may improve cardiac functions via mechanisms of immunomodulation, neovascularization, endogenous repair, inhibition of fibrosis, and proliferation of existing cardiomyocytes (Bagno et al., 2018). The majority of these studies demonstrate that the level of direct MSC contribution to cardiomyocyte replacement is low and, therefore, unlikely to represent a therapeutically meaningful MSC mechanism of action. The secretome of MSCs modulates several key cell processes that contribute to cardiovascular protection and/or repair under different pathological conditions. Genetically modified MSCs overexpressing VEGF (Locatelli et al., 2015), hepatocyte growth factor (HGF) (Zhao et al., 2016) and interleukin 10 (IL-10) (Meng et al., 2018) have been shown to alleviate cardiac injury and therefore promote cardioprotection. To address in detail the behavior of MSCs implanted in preclinical models and their impact on the site of application, labeling and tracking methods are required (Vaegler et al., 2014).

The therapeutic efficiency of human MSC-extracellular vesicles (MSC-EVs) has been observed in preclinical animal models and across many diseases and injuries (Gowen et al., 2020). CD39-expressing CD4^+^ Th1 cells initiated adenosine-related apoptosis after internalizing BM-MSC-derived exosomes (Exos) in an animal GvHD model (Amarnath et al., 2015). MSC-EVs can reduce clinical symptoms in murine models of osteoarthritis and rheumatoid arthritis. Both MSC-derived exosomes and microparticles (Wilson et al., 2019a) exerted an anti-inflammatory role on lymphocytes independent of MSC priming. In delayed-type hypersensitivity (DTH), a dose-dependent anti-inflammatory effect of MPs and Exos was observed, while in collagen-induced arthritis (CIA) models, Exos efficiently decreased clinical signs of inflammation (Cosenza et al., 2018). hUC-MSC-derived EVs may protect cardiac tissue from ischemic injury, partly by promoting angiogenesis, in a rat model of myocardial infarction (Hossein-Khannazer et al., 2021). In atopic dermatitis mouse models, intravenous administration of EV from human umbilical cord-derived MSCs (hUC-MSCs) has shown anti-atopic effects (Cho et al., 2018). In an *in vitro* AD mouse model, AT-MSC (adipose tissue-MSC)-derived EVs were shown to ameliorate the progression of beta-amyloid-induced neuronal death (Lee et al., 2018).

#### 1.4.2 Clinical Trials

MSCs were first tested as a cellular pharmaceutical agent in human subjects in 1995 by (Lazarus et al., 1995) and have since become the most clinically studied experimental cell therapy platform worldwide. After 20 years of clinical trials, MSCs have earned an excellent safety record but are still only approved for use in Canada, New Zealand, Japan, South Korea and Europe due to a lack of consistent efficacy outcomes. The obligation to register clinical studies before the start of recruitment, requested by the International Committee of Medical Journal Editors (ICJME), provides up-to-date data on ongoing clinical studies. Currently, while preparing this review, there were 1,228 clinical applications for several diseases that have been registered within the database of http://clinicaltrials.gov. Among 1,228 recorded trials, more than 485 were identified as ongoing trials. There were 370 completed clinical trials, of which 272 were in early phase I, phase I or phase II, and 28 studies advanced to phase III and IV. At the same time, 392 studies were classified as suspended, withdrawn, completed or of unknown status. Almost the majority of MSC-based clinical trials are still in phase I and II, where only a small number of trials are in phase III. In general, many of the completed clinical trials showed the efficacy of MSC-based therapies in several conditions, especially in heart diseases/failure, ischemia, arthritis, collagen diseases, infarction, joint diseases, osteoarthritis and many others, in addition to their safe administration. However, many aspects regarding MSC therapy should be deeply characterized, on the one hand, because of their broad spectrum of therapeutic potentials and, on the other hand, because their long-term follow-up safety with outcomes is not yet determined. Of all clinical trials using MSCs, the main indications are musculoskeletal diseases, with 203 registered studies, 146 trials for central nervous system diseases, 146 trials for immune system diseases, 139 for wounds and injuries, 130 for collagen diseases, 130 for rheumatic diseases, 128 for joint diseases, 127 for arthritis, 127 for vascular diseases, 123 for ischemia, 118 for respiratory tract diseases, 112 for digestive system diseases and 112 for gastrointestinal diseases.

A small part of the MSC clinical trials started using MSC-derived exosomes instead of MSCs themselves. Clinical trial number NCT: NCT04276987 used MSC-Exos for their advantages over MSCs for treating severe patients hospitalized with novel coronavirus pneumonia (NCP). Twenty-four patients were enrolled in this study (phase I); the patients received MSC-Exos derived from allogenic adipose tissue by aerosol inhalation. Based on this study, another clinical trial was performed; the patients were divided into three groups: Group 1 received Exo type I, Group 2 received Exo type II and Group 3 received a placebo. This is a combination of phase I and phase II, enrolling 30 patients (10 for each group), under the number NCT: NCT04491240. Patients also received the drug by aerosol inhalation. The results for these trials were promising, and safe administration was observed with no side effects.

Coronavirus disease (*COVID*-*19*), the ongoing pandemic, is a disease caused by the coronavirus. One of the studied treatments for severe cases is the use of exosomes derived from bone marrow MSCs (ExoFlo™). A single dose of 15 ml of ExoFlo was intravenously (IV) administered to 24 COVID-19-positive patients, and the results were promising (Sengupta et al., 2020). Several other studies suggested the beneficial and safe use of MSCs in treating severe conditions of COVID-19 patients (Golchin et al., 2020). Recently, a study evaluated the clinical outcomes of severe/critically severe COVID-19 patients (210) being treated with UC-MSCs (1–2 × 10^6^ per kilogram) from October 15, 2020 until April 25, 2021 in Turkey. UC-MSCs demonstrated safety with high potential when used as an added therapeutic treatment for severe COVID-19 patients (Ercelen et al., 2021).

Because of their therapeutic properties, the number of clinical trials using MSCs will significantly increase. Table 1 and Table 2, summarizing the clinical trials evaluating MSCs or their secretome, have been included. Data were extracted on June 18, 2021 from www.clinicalTrials.gov, using the terms “mesenchymal stem/stromal cells” or “MSC EVs,” and downloaded into an XML file. The data include the identifier for each trial (NCT number), clinical phase, recruitment status, location, start date, sponsor, gender, age and enrollment. We manually extracted additional information on disease, cell source, match (autologous vs. allogenic), route of administration, dose, cell expansion passage, conditioning and study results that could not be downloaded directly from ClinicalTrials.gov from individual trial records. Data for all of these categories were not found in many cases but were collected when possible. Doses in ClinicalTrials.gov are not reported systematically and were found either as the total numbers of cells/patient or as the number of cells/kg. While many clinical studies are in the recruitment and active phases, many of these terminate without producing a significant publication.

**TABLE 1:** Summary of the clinical trials evaluating MSCs.

NCT Number	Condition	Phases	Type of product	Match	Route of administration	Dose (10^6^ cells)	Cell expansion passage	Preconditioning	Status	Gender	Age (years)	Enrollment	Start date	Location	Funded bys	Study results
NCT04919135	Frailty	Phase 1|Phase 2	UC-MSCs	Allogenic	Intravenous	Not provided	—	—	Not yet recruiting	All	60–85	44	July 1, 2021	Not provided	Other	Not available
NCT04453111	Osteoarthritis	Phase 1|Phase 2	BM-MSCs, P-MSCs	Autologous, Allogenic	intra-articular injection	Not provided	—	—	Recruiting	All	18–75	45	January 2, 2020	Ukraine	Industry|Other	Not available
NCT03840343	Diabetic Kidney Disease	Phase 1	AD-MSCs	Autologous	intra-arterial	Not provided	—	—	Recruiting	All	45–75	30	October 23, 2019	United States	Other	Not available
NCT04433104	Chronic Obstructive Pulmonary Disease	Phase 1|Phase 2	UC-MSCs	Allogenic	Intravenous	1/kg	—	—	Recruiting	All	40–75	40	June 9, 2020	Vietnam	Other	Not available
NCT04759105	Chronic Low Back Pain	Phase 2	BM-MSCs	Autologous	intradiscal injection	15/disc	—	—	Recruiting	All	18–65	52	November 17, 2020	Italy	Other	Not available
NCT03874572	Dry Mouth Syndrome	Phase 1	AD-MSCs	allogeneic	Intraglandular injection	Not provided	—	—	Active, not recruiting	All	18–75	10	March 18, 2019	Denmark	Other	Not available
NCT03478215	Kidney Transplantation	Phase 2	MSCs	Autologous	Intravenous	1 to 3/kg	—	—	Recruiting	All	18–65	24	February 2016	United States	Other	Not available
NCT04356287	Systemic Sclerosis	Phase 1|Phase 2	UC-MSCs	Allogenic	Intravenous	1/kg	—	—	Not yet recruiting	All	18 and older	18	July 2021	Not provided	Other	Not available
NCT03876197	Radiation-induced Hyposalivation and Xerostomia	Phase 1|Phase 2	AD-MSCs	Autologous	Intraglandular injection	Not provided	—	—	Enrolling by invitation	All	18–99	30	August 1, 2020	Denmark	Other	Not available
NCT01586312	Osteoarthritis	Phase 1|Phase 2	BM-MSCs	Allogenic	intra-articular injection	40	2	—	Completed	All	18–75	30	April 2012	Spain	Industry|Other	Allogeneic MSC therapy may be a valid alternative for the treatment of chronic knee osteoarthritis. doi: 10.1097/TP.0000000000000678
NCT01860417	Degenerative Disc Disease	Phase 1|Phase 2	BM-MSCs	Allogenic	intradiscal injection	25/disc	expanded *ex vivo* for 3–4 weeks	—	Completed	All	18–75	25	April 2013	Spain	Industry|Other	Quick and significant improvement in algofunctional indices versus the controls. doi: 10.1097/TP.0000000000001484
NCT03389919	Graft Failure	Phase 3	MSCs	Not provided	Intraosseous	Not provided	—	—	Recruiting	All	18 and older	20	January 1, 2017	Russian Federation	Other	Not available
NCT02195323	Chronic Kidney Disease	Phase 1	BM-MSCs	Autologous	Intravenous	Not provided	—	—	Completed	All	25–60	7	April 2014	Iran	Other	Not available
NCT04325594	Chronic Heart Failure	Phase 2	UC-MSCs	Allogenic	intracoronary injection	10	—	—	Enrolling by invitation	All	18 and older	60	March 1, 2020	Kazakhstan	Other	Not available
NCT02495766	Multiple Sclerosis	Phase 1|Phase 2	BM-MSCs	Autologous	Intravenous	Not provided	—	—	Completed	All	18–60	8	May 11, 2015	Spain	Other	Not available
NCT02384018	Islet Autograft	Phase 1	BM-MSCs	Autologous	Intravenous	20 or 50 or 100	—	—	Active, not recruiting	All	18–69	42	December 2014	United States	Other|NIH	Not available
NCT02408432	Heart Failure	Phase 1	BM-MSCs	Allogenic	Intravenous	Not provided	—	—	Recruiting	All	18–80	45	January 11, 2016	United States	Other|NIH	Not available
NCT03795974	Cerebral Palsy	Phase 2	UC-MSCs	Allogenic	intrathecal injection	Not provided	—	—	Unknown status	All	avr-14	108	July 23, 2017	Iran	Other	Not available
NCT02962661	Heart Failure	Phase 1	BM-MSCs	Allogenic	Intravenous	Not provided	—	—	Recruiting	All	18–80	72	July 18, 2020	United States	Other|NIH	Not available
NCT03745417	Psoriasis	Phase 1|Phase 2	UC-MSCs	Allogenic	Intravenous	2/kg	—	—	Not yet recruiting	All	18–65	5	August 31, 2021	China	Other	Not available
NCT02801890	Peritoneal Fibrosis	Phase 1|Phase 2	AD-MSCs	Autologous	Intravenous	1/kg	—	—	Completed	All	18–70	10	August 2015	Iran	Other	Not available
NCT01275612	Solid Organ Cancers	Phase 1	MSCs	Not provided	Intravenous	1/kg	—	—	Withdrawn	All	18–80	0	November 2010	Italy	Other	Not available
NCT00781872	Multiple Sclerosis	Phase 1|Phase 2	BM-MSCs	Autologous	IV, Intrathecal	1/kg	40–60 days	—	Completed	All	35–65	24	October 2006	Not provided	Other	Transplantation of MSCs in patients with MS and ALS is a clinically feasible and relatively safe procedure and induces immediate immunomodulatory effects. doi:10.1001/archneurol.2010.248
NCT04869761	Chronic Kidney Diseases	Phase 1	AD-MSCs	Allogenic	Intravenous	150	—	—	Not yet recruiting	All	40–80	40	May 2021	United States	Other	Not available
NCT03265613	Psoriasis	Phase 1|Phase 2	AD-MSCs	Allogenic	Intravenous	0,5/kg	—	—	Active, not recruiting	All	18–65	7	September 24, 2017	China	Other	Not available
NCT03392311	Psoriasis	Phase 1|Phase 2	AD-MSCs	Allogenic	Intravenous	2/kg	—	—	Enrolling by invitation	All	18–65	8	August 17, 2019	China	Other	Not available
NCT02646007	Kienböck’s Disease	Phase 1	BM-MSCs	Autologous	Injection	Not provided	—	—	Unknown status	All	18–65	30	November 2015	Iran	Other	Not available
NCT03042143	Acute Respiratory Distress Syndrome	Phase 1|Phase 2	UC-CD362 enriched MSCs	Not provided	Infusion	100, 200, 400	—	—	Recruiting	All	16 and older	75	January 7, 2019	United Kingdom	Other	Not available
NCT00644410	Hart Failure	Phase 1|Phase 2	BM-MSCs	Autologous	intramyocardial injection	77,5	2	—	Completed	All	30–80	59	September 2008	Denmark	Other	Expanded MSCs were safe and improved myocardial function in patients with severe ischaemic heart failure. doi:10.1093/eurheartj/ehv136
NCT04519671	Crohn’s Disease	Phase 1|Phase 2	BM-MSCs	Allogenic	Direct injection	75	—	—	Recruiting	All	18–75	40	November 19, 2020	United States	Other	Not available
NCT04785027	Psoriasis	Phase 1|Phase 2	AD-MSCs	Allogenic	Intravenous	2/kg	—	—	Recruiting	All	18–65	16	March 17, 2021	China	Other	Not available
NCT04371393	Covid -19 Acute Respiratory Distress Syndrome	Phase 3	remestemcel-L	Not provided	Intravenous	2/kg	—	—	Active, not recruiting	All	18 and older	223	April 30, 2020	United States	Other|Industry|NIH	Not available
NCT03325322	Chronic Kidney Disease	Phase 2	Not provided	Not provided	Not provided	Not provided	—	—	Recruiting	All	40–80	30	January 2, 2018	United States	Other	Not available
NCT02940418	Diabetes	Phase 1	AD-MSCs	Allogenic	Intravenous	1/kg, 10/kg	5	—	Recruiting	All	18–35	20	February 19, 2017	Jordan	Other	Not available
NCT00395200	Multiple Sclerosis	Phase 1|Phase 2	BM-MSCs	Autologous	Intravenous	2/kg	—	—	Completed	All	18–65	10	July 2008	United Kingdom	Other	The evidence of structural, functional, and physiological improvement after treatment in some visual endpoints is suggestive of neuroprotection. doi: 10.1016/S1474-4422(1170305-2)
NCT03042572	No-option Severe Limb Ischemia	Phase 2|Phase 3	BM-MSCs	Allogenic	Intramuscular injection	150	—	—	Not yet recruiting	All	18 and older	60	December 2018	Netherlands	Other	Not available
NCT02685098	Amputation	Phase 1	BM-MSCs	Allogenic	—	Not provided	—	—	Recruiting	All	40–90	16	January 2017	United States	Other	Not available
NCT04447833	Covid -19 Acute Respiratory Distress Syndrome	Phase 1	BM-MSCs	Allogenic	Intravenous	1/kg, 2/kg	—	—	Active, not recruiting	All	18–65	7	June 17, 2020	Sweden	Other	Not available
NCT01468064	Ischemic Stroke	Phase 1|Phase 2	BM-MSCs and EPCs	Autologous	Intravenous	2,5/kg	33.75 days	—	Completed	All	18–80	20	November 2011	China	Other|Industry	Autologous transplantation of EPCs appears to improve long-term safety in acute cerebral infarct patients. doi: 10.1002/sctm.18-0012
NCT02379442	Graft-Versus-Host-Disease	Phase 1|Phase 2	BM-MSCs	Allogenic	Intravenous	2 × 1/kg, 12 doses	—	—	Terminated	All	4–99	1	February 23, 2015	United States	NIH	Not available
NCT02893306	Diabetes	Phase 2	BM-MSCs	Allogenic	Intravenous	2–3/kg	—	—	Unknown status	All	18 and older	10	March 2012	Chile	Other	Not available
NCT02017912	Amyotrophic Lateral Sclerosis	Phase 2	BM-MSC-NTF	Autologous	Intramuscular and intrathecal	Not provided	—	induced to secrete NTFs by a medium based approach	Completed	All	18–75	48	May 2014	United States	Industry	Not available
NCT03164083	Osteoarthritis	Phase 2	BM-MSCs, SVF	Autologous	intra-articular injection	Not provided	—	—	Withdrawn	All	25–65	0	November 10, 2019	Iran	Other	Not available
NCT03015623	Acute Kidney Injury	Phase 1|Phase 2	MSCs	Allogenic	integrated into the renal replacement circuit	250–750	—	—	Active, not recruiting	All	18 and older	24	June 20, 2017	United States	Industry	Not available
NCT03211793	Ulcers of Systemic Sclerosis	Phase 1|Phase 2	BM-MSCs	Allogenic	Intramuscular	50	—	—	Unknown status	All	18 and older	20	November 2018	Netherlands	Other	Not available
NCT04466098	Covid -19Acute Respiratory Distress Syndrome	Phase 2	MSCs	Not provided	Intravenous	300	—	—	Active, not recruiting	All	18–80	9	July 30, 2020	United States	Other	Not available
NCT02274428	Premature Infants	Phase 1	UC-MSCs	Not provided	—	Not provided	—	—	Completed	All	23–34	9	October 2014	Korea	Other	Not available
NCT00927355	Effect of Thiazolidinediones on Human Bone	Not Applicable	Pioglitazone	Not provided	—	Not provided	—	—	Completed	All	18–80	10	April 2009	United States	Other	Insights into the deleterious effects of TZDs on bone quality in diabetics and support a model in which TZD-induced adipogenesis may be a significant influencing factor on osteoblast differentiation and function. doi: 10.1016/j.trsl.2012.08.006
NCT04247945	Allogeneic Hematopoietic Stem Cell Transplantation	Phase 2|Phase 3	MSCs	Not provided	—	Not provided	—	—	Recruiting	All	up to 65 Years	120	February 1, 2020	China	Other	Not available
NCT01144962	Crohn’s Disease	Phase 1|Phase 2	BM-MSCs	Allogenic	Local injection	10–30-90	2	—	Completed	All	18 and older	21	June 2010	Netherlands	Other	Local administration of allogeneic MSCs was not associated with severe adverse events in patients with perianal fistulizing Crohn’s disease. Injection of 3 × 10^7^ MSCs appeared to promote healing of perianal fistulas.doi: 10.1053/j.gastro.2015.06.014
NCT03102879	Periapical Periodontitis	Not Applicable	Encapsulated UC-MSCs in a biological scaffold	Allogenic	Conventional Root Canal Treatment	Not provided	—	—	Completed	All	16–58	36	September 23, 2016	Chile	Other	Safety and efficacy evidence of the endodontic use of allogenic umbilical cord mesenchymal stem cells encapsulated in a plasma-derived biomaterial. doi: 10.1177/0022034520913242
NCT01739504	Osteoarthritis	Not Applicable	AD-MSCs	Autologous	Intra articular	—	—	—	Terminated	All	18–80	10	March 1, 2014	United States	Industry	Not available
NCT03298399	Sickle Cell Disease	Phase 1	BM-MSCs	Autologous	Intravenous	2/kg	28 days	—	Withdrawn	All	déc-40	0	December 21, 2017	Not provided	Other|NIH	Not available
NCT02421484	Septic Shock	Phase 1	BM-MSCs	Allogenic	Intravenous	0,3/kg, 1/kg, 3/kg	—	—	Completed	All	18 and older	9	May 2015	Canada	Other	The infusion of freshly cultured allogenic bone marrow-derived MSCs, up to a dose of 3 million cells/kg (250 million cells), into participants with septic shock seems safe. doi: 10.1164/rccm.201705-1006OC
NCT02145923	Neutropenic Enterocolitis	Phase 1|Phase 2	BM-MSCs	Allogenic	Intravenous	1,5–2/kg	—	—	Unknown status	All	18–65	16	May 2014	Russian Federation	Other	Not available
NCT03818737	Osteoarthritis	Phase 3	BM-MSCs, AD-MSCs, UC-MSCs	Autologous	Orthobiologic injection	Not provided	—	—	Active, not recruiting	All	40–70	480	March 28, 2019	United States	Other	Not available
NCT02033525	Degenerative Meniscus Injury	Phase 1|Phase 2	XCEL-M-ALPHA	Autologous	—	Not provided	—	—	Completed	All	40–60	20	January 31, 2014	Spain	Other	Not available
NCT02209311	Maxilla Alveolar Process Reconstruction	Phase 1|Phase 2	Oral mucosa-MSCs-tissue engineered construction	Autologous	Implantation	Not provided	3–4 weeks	—	Unknown status	All	20–60	12	September 2014	Russian Federation	Other	Not available
NCT04445220	Acute Kidney Injury	Phase 1|Phase 2	MSCs (SBI-101)	Allogenic	Integration	250–750	—	—	Recruiting	All	18 and older	22	November 19, 2020	United States	Industry	Not available
NCT01849159	Pulmonary Emphysema	Phase 1|Phase 2	BM-MSCs	Allogenic	Intravenous	200	—	Hypoxia (1% pxygen)	Withdrawn	All	35–75	0	March 2014	Russian Federation	Other	Not available
NCT04629833	Steroid-Refractory Acute Graft-Versus-Host Disease	Phase 3	MSCs-MC0518	Not provided	Intravenous	1–2/kg	—	—	Not yet recruiting	All	12 and older	210	June 30, 2021	France, Germany, Poland, Spain	Industry	Not available
NCT04007081	XRT Induced Xerostomia	Not Applicable	BM-MSCs	Autologous	Salivary Gland Autotransplantation	50	2 weeks	—	Completed	All	18–89	12	October 18, 2019	United States	Other	Not available
NCT03643614	Post-Irradiation Vaginal-Rectal Fistula	Phase 1	AD-MSCs	Autologous	Direct injecton	Not provided	—	—	Completed	Female	20–75	16	August 1, 2017	Russian Federation	Other	Not available
NCT02630836	Hip Fracture	Phase 1|Phase 2	BM-MSCs	Allogenic	Surgical treatment	Not provided	—	—	Withdrawn	All	70–85	0	December 2015	Not provided	Other	Not available
NCT02323477	Myocardial Infarction	Phase 1|Phase 2	UC-MSCs, BM-MSCs	Allogenic, Autologous	peri-infarct areas	UC-MSCs (23), BM-MSCs (700)	—	—	Terminated	Male	30–80	46	February 2, 2015	Turkey	Other|Industry	Intramyocardial administration of HUC-MSCs in combination with CABG displayed higher scores in reducing the scar tissue and restoration of ventricular wall functions compared with autologous BM-MNCs. doi: 10.1007/s12015-015-9601-0
NCT04173650	Dystrophic Epidermolysis Bullosa	Phase 1|Phase 2	Exosomes from BM-MSCs (AGLE-102)	Allogenic	—	2 doses	—	—	Not yet recruiting	All	6 and older	10	January 2022	—	Industry	Not available
NCT04366063	Covid-19 Related Acute Respiratory Distress Syndrome	Phase 2|Phase 3	MSCs + Evs	—	Intravenous	2 MSC infusions (100) + 2 EVs infusions	—	—	Recruiting	All	18–65	60	June 6, 2020	Iran	Other	Not available

**TABLE 2:** Summary of the clinical trials evaluating the secretome of MSCs.

NCT Number	Condition	Phases	Type of product	Match	Route of administration	Dose (10^6^ cells) or MV or Exo	Cell expansion passage	Preconditioning	Status	Gender	Age (years)	Enrollment	Start date	Location	Funded bys	Study results
NCT02138331	Type 1 Diabetes	Phase 2|Phase 3	Cell free [UC-MSCs-MV and Exo]	NA	Intravenous	Exo or MV: from SN of 1.2–1.5/kg (MSCs)	NA	NA	Unknown status	All	18–60	20	April 2014	Egypt	Other	NA
NCT04798716	Covid -19 Acute Respiratory Distress Syndrome	Phase 1|Phase 2	Cell free [Perinatal MSCs-Exo]	NA	Intravenous	Tree groups: Escalating Dose: 2/4/8; 8/4/8; 8/8/8 (x10^3^/mL)	NA	NA	Not yet recruiting	All	18 and older	55	April 2021	United States	Industry	NA
NCT03437759	Macular Holes	Early Phase 1	Cell free [UC-MSCs-Exo]	NA	Intravitreal	20–50 µg MSC-Exo	NA	NA	Active, not recruiting	All	Up to 80	44	March 1, 2017	China	Other	NA
NCT04276987	Covid -19 Pneumonia	Phase 1	Cell free [AD-MSCs-Exo]	Allogenic	Aerosol inhalation	5 x (2 × 10^8^ nv/mL)	NA	NA	Completed	All	18–75	24	February 15, 2020	China	Other|Industry	NA
NCT04356300	Multiple Organ Dysfuntion Syndrome	Not Applicable	Cell free [UC-MSCs-Exo]	NA	Intravenous	(14x) 150 mg/day	NA	NA	Not yet recruiting	All	20–80	60	September 1, 2020	NA	Other	NA
NCT04388982	Alzheimer Disease	Phase 1|Phase 2	Cell free [AD-MSCs-Exo]	Allogenic	Nasal drip	5–10–20 µg (2x/W) for 12 W	NA	NA	Recruiting	All	50 and older	9	July 1, 2020	China	Other|Industry	NA
NCT03384433	Ischemic Stroke	Phase 1|Phase 2	Cell free [MSCs-Exo]	Allogenic	Stereotaxis/Intraparanchymal	NA	NA	Exosome enriched by miR-124	Recruiting	All	40–80	5	April 17, 2019	Iran	Other	NA
NCT04313647	Healthy Volunteers: Tolerance test	Phase 1	Cell free [AD-MSCs-Exo]	Allogenic	Aerosol inhalation	2-4-8–12–16–20 (x10^8^ nv/3 ml)	NA	NA	Recruiting	All	18–45	27	March 16, 2020	China	Other|Industry	NA
NCT04850469	Severely Infected Children	NA	NA	NA	NA	NA	NA	NA	Not yet recruiting	All	Up to 18	200	January 1, 2022	China	Other	NA
NCT04173650	Dystrophic Epidermolysis Bullosa	Phase 1|Phase 2	Cell free [BM-MSCs-Exo]	Allogenic	Local	6 ascending doses	NA	NA	Not yet recruiting	All	6 and older	10	April 2021	NA	Industry	NA
NCT03608631	Metastatic Pancreas Cancer	Phase 1	Cell free [MSCs-Exo with KRAS G12D siRNA]	NA	Intravenous	(15–20 min on days 1, 4, and 10); 3 courses/14 days	NA	KrasG12D siRNA-loaded MSCs-Exo	Recruiting	All	18 and older	28	January 27, 2021	United States	Other	NA
NCT04602442	Covid--19 Pneumonia	Phase 2	Cell free [MSCs-Exo]	NA	Aerosol inhalation	2x (0,5–2 x10^10^ nanoparticles)/10 days	NA	NA	Enrolling by invitation	All	18–65	90	October 1, 2020	Russian Federation	Other	NA
NCT04491240	Covid--19 Pneumonia	Phase 1|Phase 2	Exosomes	NA	Aerosol inhalation	2x (0,5–2 x10^10^ nanoparticles)/10 days	NA	NA	Completed	All	18–65	30	July 20, 2020	Russian Federation	Other	NA
NCT03857841	Bronchopulmonary Dysplasia	Phase 1	Cell free [BM-MSCs-EVs] UNEX-42	NA	Intravenous	20–60–200 pmol EV/kg	NA	NA	Terminated	All	Up to 14 days	3	June 20, 2019	United States	Industry	NA
NCT04213248	Dry Eye	Phase 1|Phase 2	Cell free [UC-MSCs-Exo]	NA	Artificial tears	10ug/drop, (4x)/day (14 days)	NA	NA	Recruiting	All	18–70	27	February 18, 2020	China	Other	NA
Kordelas et al., 2014	Graft-Versus-Host-Disease	NA	Cell free [BM-MSCs-Exo]	Allogenic	Intravenous	1.3–3.5 × 10^10^ particles/unit; 0.5–1.6 mg/unit)	3	NA	Completed	Female	22	1	NA	Germany	—	The clinical GvHD symptoms improved significantly shortly after the start of the MSC-exosome therapy. Kordelas et al., 2014. doi:10.1038/leu.2014.41
Nassar et al., 2016	Chronic Kidney Diseases	Phase 2|Phase 3	Cell free [UC-MSCs-EVs]	Allogenic	Intravenous/Intra-arterial	100 μg EV/kg/dose (2 doses)	6	NA	Completed	All	26–44	40	NA	Egypt	Other	Administration of cell-free cord-blood mesenchymal stem cells derived extracellular vesicles (CF-CBMSCs-EVs) is safe and can ameliorate the inflammatory immune reaction and improve the overall kidney function in grade III-IV CKD patients. Nassar et al., 2016. doi 10.1186/s40824-016-0068-0
NCT04270006	Periodontitis	Early Phase 1	Cell free [AD-MSCs-Exo]	Autologous	Local	NA	NA	NA	Recruiting	All	18–50	10	February 12, 2020	Egypt	Other	NA
NCT04202783	Craniofacial Neuralgia	Not Applicable	Neonatal stem cell -Exo	—	Focused ultrasound epineural injection and Intravenous	2 x (5 ml concentrated Exo) + 45 mg Exo +15 mg Exo	NA	NA	Suspended	All	18 and older	100	December 1, 2021	United States	Other	NA
NCT04202770	Depression, Anxiety, and Dementias	Not Applicable	Cell free (amniotic fluid-MSC-Exo)	Allogenic	Focused ultrasound Intravenous	Eq 20 stem cells	NA	NA	Suspended	All	18 and older	300	December 1, 2019	United States	Other	NA
NCT04384445	COVID-19 Acute Respiratory Distress Syndrome	Phase 1|Phase 2	Amniotic fluid stem cell organicell Flow)	—	Intravenous	2–5 x 10^11^ particles/mL (3 times)	NA	NA	Recruiting	All	18 and older	20	September 8, 2020	United States	Industry	NA
NCT04223622	Osteoarthritis	—	Cell free [AD-MSCs-CM or Evs)	—	*ex vivo* Osteoarthritis model	NA	NA	NA	Not yet recruiting	All	18 and older	24	February 2020	—	Other	NA
NCT04270006	Periodontitis	Early Phase 1	Cell free [AD-MSCs-Exo]	Autologous	Local	NA	NA	NA	Recruiting	All	18–50	10	February 12, 2020	Egypt	Other	NA
NCT04134676	Chronic Ulcer Wounds	Phase 1	Cell free [WJ-MSCs-CM)	NA	Local	CM gel (for 2 w)	NA	NA	Completed	All	18–80	38	June 1, 2019	Indonesia	Other|Industry	NA
NCT04544215	Pulmonary Infection	Phase 1|Phase 2	Cell free [AD-MSCs-Exo]	Allogenic	Aerosol inhalation	8 × 10^8^ or 16 × 108 nano vesicles/3 ml	NA	NA	Recruiting	All	18–75	60	July 1, 2020	China	Other|Industry	NA
Sengupta et al	Severe COVID-19	NA	Cell free [BM-MSCs-Exo]	Allogenic	Intravenous	15 ml of ExoFlo	NA	NA	Completed	NA	18–85	27	April 2020	United States	Other	Owing to its safety profile, capacity to restore oxygenation, downregulate cytokine storm, and reconstitute immunity, ExoFlo is a promising therapeutic candidate for severe COVID-19. Sengupta et al.(2020), doi: 10.1089/scd.2020.0080

##### 1.4.2.1 Primed Mesenchymal Stem/Stromal Cells

To enhance the beneficial properties of MSCs, several priming strategies have been proposed. However, few clinical trials have reported the use of primed MSCs for better therapeutic efficacy, and inflammatory priming has not yet been clinically investigated. Using a medium-based approach, MSCs can be induced to secrete elevated levels of neurotropic factors, which have been shown to have protective effects (Gothelf et al., 2014). These cells, designated MSC-NTF cells (neurotrophic factor-secreting MSCs, also known as NurOwn™) derived from patients’ own bone marrow, have been recently used for phase I/II and phase IIa of clinical studies in patients with amyotrophic lateral sclerosis (ALS). In these studies, ALS patients were subjected to a single administration of autologous MSC-NTF cells. The data from these studies indicate that the single administration of MSC-NTF cells is safe, well tolerated and demonstrated early promising signs of efficacy (Abdul Wahid et al., 2019; Berry et al., 2019). Another option involved the culture of allogeneic BM-MSCs in hypoxic conditions (1% oxygen) to potentiate their efficacy (NCT01849159) (https://clinicaltrials.gov/ct2/show/NCT01849159) (Oh et al., 2017). A clinical study addressed the use of autologous platelet lysate (PL) to expand MSCs as a treatment for knee osteoarthrosis (KOA). Thirteen patients were enrolled in this study (phase II). The patients were divided into two groups, with one receiving autologous bone marrow-derived MSCs alone and the other receiving autologous bone marrow MSCs primed with platelet lysate and with both infused by intraarticular injections (https://clinicaltrials.gov/ct2/show/NCT02118519). Preliminary data concluded the safety of injections of MSCs for knee osteoarthritis patients; efficacy was also established for more than 2 years of follow-ups (Al-Najar et al., 2017). Umbilical cord-derived MSCs (UC-MSCs) have also demonstrated safety and efficacy in clinical trials of several diseases and conditions, such as RA (Wang et al., 2019). Clinical trials, such as clinical trial number NCT01547091, infused umbilical cord MSCs (UC-MSCs) intravenously (IV) several times for an interval of time. In that clinical trial (phase I/II), 200 patients were included, where some received MSC treatment, others received disease-modified antirheumatic drugs (DMARDs) and others received a combination of MSCs with DMARDs.

##### 1.4.2.2 Approved Mesenchymal Stem/Stromal Cells Products

Several companies have or are in the process of commercializing MSC-based therapies. Despite the amount of research that has been conducted and the number of clinical trials, there are few approved products (Table 3). The European Medicines Agency (EMA) is responsible for the scientific evaluation of centralized marketing authorization applications (MAAs). Once granted by the European Commission, the centralized marketing authorization is valid in all European Union (EU) member states, Iceland, Norway and Liechtenstein (https://www.ema.europa.eu). The US Food and Drug Administration (FDA) has the authority to regulate regenerative medicine products, including stem cell products and exosome products (https://www.fda.gov/). Currently, the only stem cell products that are FDA-approved for use in the United States consist of blood-forming stem cells (also known as hematopoietic progenitor cells) that are derived from umbilical cord blood. In the United States, stem cell products and exosome products should be carefully verified before use by considering FDA approval or being studied under an Investigational New Drug Application (Berry et al., 2019), which is a clinical investigation plan submitted and allowed to proceed by the FDA. Delivering a safe and effective product is key, and effective guidance from organizations such as the FDA and the ARM (Alliance for Regenerative Medicine) will ease and accelerate the translation of MSC technologies from the bench top to the bedside (Olsen et al., 2018).

**TABLE 3:** MSC and MSC progenitors- based products with marketing approval for clinical application worldwide.

MSC-product	Indication	MSC type	Company	Country (Marketing approval year)	Regulatory agency
Alofisel	Complex perianal fistulas in Crohn’s disease	Allogeneic AD-MSCs	Takeda Pharma	Europe (2018)	EMA
Allostem	Bone regeneration	Allogeneic AD-MSCs	AlloSource	United States (2010)	Regulated under CFR 1,270, 1,271 as a human tissue. Do not require pre-market approval from the FDA. (Ref. 1; Ref. 2)
Cartistem	Osteoarthritis	Allogeneic UC-MSCs	Medipost Co., Ltd	South Korea (2012)	MFDS
Grafix	Acute/chronic wounds	Allogeneic placental membrane, incuding MSCs	Osiris Therapeutics	United States (2011)	Products marketed as human cells, tissues, and cellular and tissue-based products (“HCT/Ps”), as defined by the US FDA, that are regulated solely under Section 361 of the Public Health Service Act (“361 HCT/Ps”), and consequently, do not require pre-market approval from the FDA. https://fintel.io/doc/sec-osir-osiris-therapeutics-10k-2019-march-15-17970
					other sources: http://www.osiris.com/grafix/certifications/
Prochymal (remestemcel-L)	GvHD	Allogeneic BM-MSCs	Osiris Therapeutics Inc./Mesoblast	Canada (2012)	Health Canada (expired date protection 2020)
				New Zealand (2012)	MEDSAFE (Approval lapsed)
OsteoCel	Orthopaedic repair	Allogeneic BM-MSCs	NuVasive	United States (2005)	Regulated under CFR 1270, 1271 as a human tissue. Do not require pre-market approval from the FDA. (Ref. 1, Ref. 2)
Bio4 (formerly OvationOS)	Bone repair and regeneration	Bone-forming osteoblasts, osteoprogenitor cells and MSCs	Osiris Therapeutics	United States (2014)	Do not require pre-market approval from the FDA: US FDA regulations for tissue management. US FDA 21 CFR 1271
			Stryker		https://fintel.io/doc/sec-osir-osiris-therapeutics-10k-2019-march-15-17970
Temcell HS	GvHD	Allogeneic BM-MSCs	JCR Pharmaceuticals	Japan (2015)	PMDA
Trinity Evolution	Orthopaedic repair	Allogeneic BM-MSCs	Orthofix	United States (2019)	Do not require pre-market approval from the FDA: US FDA regulations for tissue management. US FDA 21 CFR 1271 (Ref 2)
Trinity Elite	Orthopaedic repair	Allogeneic BM-MSCs	Orthofix	United States (2013)	Regulated under CFR 1270, 127cer1 as a human tissue (Ref1)
QueenCell	Subcutaneous tissue defects	Autologous AD-MSCs	Anterogen Co., Ltd.	South Korea (2010)	MFDS
Ossron	Bone regeneration	Autologous BM-MSCs	Sewon Cellontech CO., Ltd.	South Korea (2009)	MFDS
Obnitix	GvHD	Allogeneic BM-MSCs	Medac	Germany	NA
Stempeucel	Critical limb ischemia	allogeneic BM-MSCs	Stempeutics	India (2016)	DCGI
			Research		
Neuronata-R	Amyotrophic lateral sclerosis	autologous BM-MSCs	Corestem, Inc.	South Korea (2014)	MFDS
Cellgram-AMI	Myocardial infarction	autologous BM-MSCs	Pharmicell Co., Ltd.	South Korea (2011)	MFDS
Cupistem	Crohn’s fistula	autologous AD-MSCs	Anterogen Co., Ltd.	South Korea (2012)	MFDS
Stemirac	Spinal cord injury	Autologous BM-MSCs	Nipro Corp	Japan (2018)	PMDA
Cellentra VCBM	Orthopaedic repair	Allogeneic BM-MSCs	Biomet Inc	United States (2012)	US FDA regulations for tissue management. US FDA 21 CFR 1271 (Ref. 1; Ref. 3)
HiQCell	Osteoarthritis/tendonitis	Autologous adipose stromal vascular fraction	Regeneus Ltd. (ASX:RGS)	Australia (2013)	NA
LiquidGen	Bone repair	Allogeneic BM-MSCs	Skye Orthobiologics LLC	United States	NA
CardioRel	Myocardial infarction	Autologous MSCs	Reliance life sciences	India (2010)	NA
Adipocel	Crohn’s disease	Autologous AD-MSCs	Anterogen Co., Ltd.	South Korea (2007)	NA
Autostem	Subcutaneous fat loss area	Autologous AD-MSCs	Cha biotech	South Korea (2010)	NA
MesestroCell	Osteoarthritis and knee joint arthritis	Autologous BM-MSCs	Cell Tech Pharmed	Iran (2018)	NA

Across the world, there are 10 approved MSC-based therapies, including Alofistel for Crohn’s disease (approved in Europe); Prochymal for GvHD (approved in Canada and New Zealand); Temcell HS Inj for GvHD (approved in Japan); Queencell for subcutaneous tissue defects; Cupistem for Crohn’s fistula, Neuronata-R for amytrophic lateral sclerosis and Cartistem for knee articular cartilage defects (all approved in South Korea); Stemirac for spinal cord injury (approved in Japan); Stempeucel for critical limb ischemia (approved in India); and Cellgram-AMI for acute myocardial infarction (approved in South Korea).

BM-MSCs from a healthy adult donor were used to produce TEMCELL, the first world therapeutic product using MSCs that was approved in Japan in September 2015 for the treatment of acute GvHD (Okada et al., 2017). One of the rare clinical trials in phase III is the use of allogeneic adipose tissue-derived MSCs for complex perianal fistulas in Crohn’s disease (clinical trial number NCT: NCT01541579). The TiGenix/Takeda phase III clinical trial that evaluates the use of MSCs for complex perianal fistulas in Crohn’s disease (CD) is arguably the most successful late-stage MSC trial to date (NCT01541579). In 2018, MSCs received European approval to be used to treat patients with Crohn’s-related enterocutaneous fistular disease (Panes et al., 2018). The approved pharmaceutical drug, Alofisel, is derived from adipose allogeneic MSCs. According to the indicated study, adult CD patients with treatment-refractory, draining, complex perianal fistulas treated with allogeneic AT-MSCs (Alofistel) showed good remission, demonstrating the potential of MSCs to substantially improve the standard of care in chronic illnesses such as CD. The study is one of the clinical trials performed to test Darvadstrocel (Alofisel), which is still called Cx601 (suspension of adipose-derived MSCs). This randomized and double-blind study enrolled 212 patients who received a single dose of 120 million MSCs (Cx601) or 24 ml saline solution (placebo) by intralesional injection. The results were promising, and it was concluded that Cx601 was an effective and safe treatment for perianal fistulas in patients with Crohn’s disease (Panes et al., 2016). Another clinical trial, active at present, is testing Darvadstrocel and is registered under the number NCT: NCT03706456. The study is supposed to be completed on January 31, 2023; it enrolled 22 participants, and it will include a follow-up period of 52 weeks after study product administration and a long-term follow-up period from week 52 to week 156.

## 2 Pre-Clinical Challenges

**FIGURE 2:**
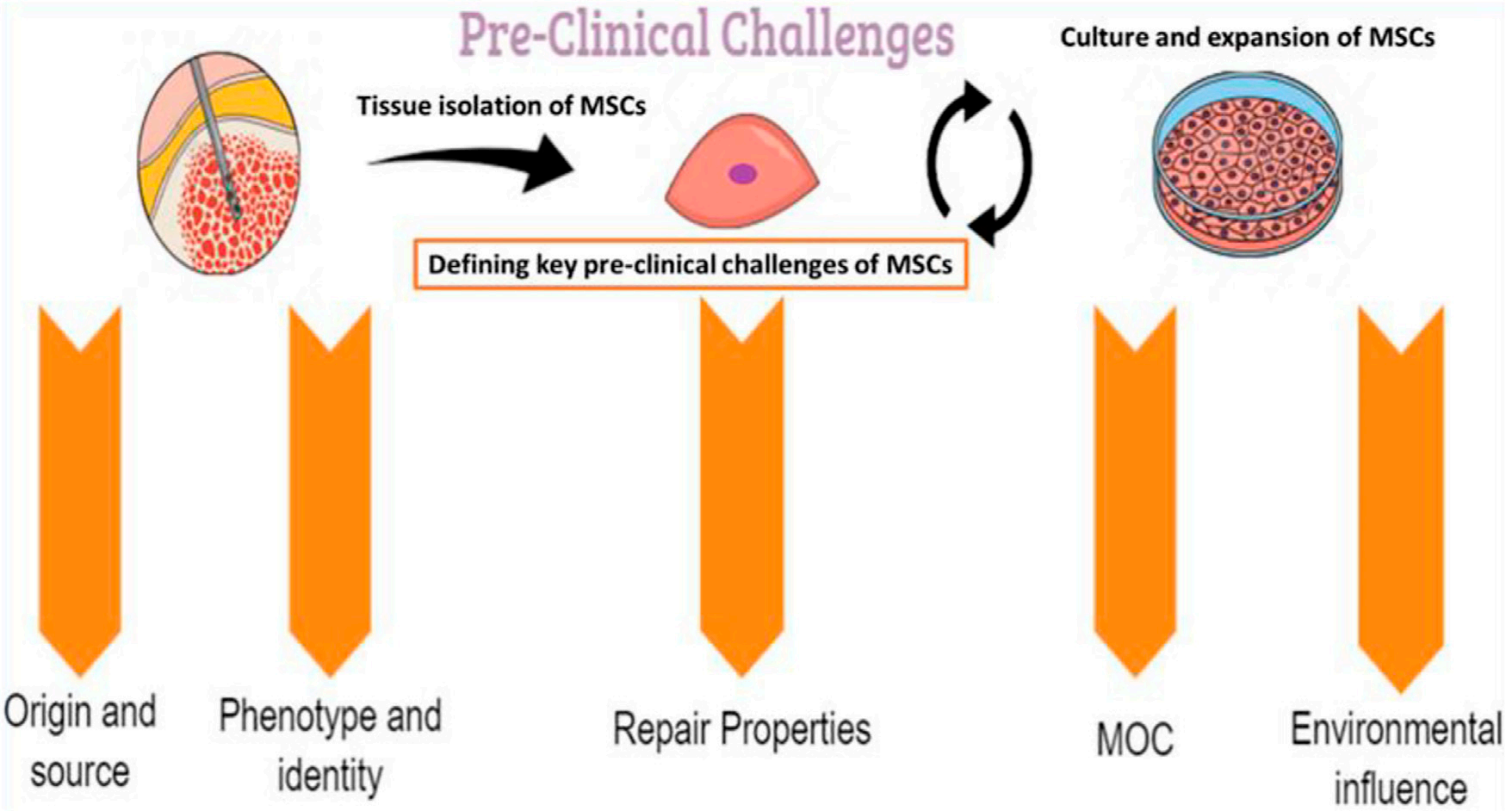
The pre-clinical challenges linked to MSCs. MOC: mechanisms of action.

Several preclinical challenges may influence the therapeutic use of MSCs and should be well identified and characterized (Figure 2).

### 2.1 Tissue Sources of Mesenchymal Stem/Stromal Cells

MSCs are virtually present in all tissues and share some characteristics, such as similar shape, phenotype and functions (Song et al., 2020a). Thus, several sources are reported to allow the isolation of MSCs. Even when the expansion step is successful and a high number of cells are transplanted during the procedure, the cells frequently have very reduced viability and low engraftment in the recipient tissue (Haque et al., 2015). These alterations have led to distinct biological properties of MSC populations, which may partly explain the differences in the outcomes of clinical trials with distinct MSCs. It is well established that over culture passages, MSCs enter a state of replicative senescence after 20–30 cell divisions (Martin et al., 2016). During this process, MSC morphology changes from relatively small spindle-shaped cells to larger and flattened cells, with typically more pronounced actin cytoskeleton fibers. Thus, over the passages, MSCs isolated *in vitro* more often resemble a cellular mixture with variable properties, resulting from intrinsic and extrinsic influences in addition to inherent disparities related to different sources and donors (Naji et al., 2019).

#### 2.1.1 Bone Marrow

MSCs have traditionally been derived from bone marrow for clinical trials and *in vitro* research. The isolation and expansion of bone marrow-derived MSCs involves the aspiration of the iliac crest followed by the isolation of the mononuclear cell fraction by density-gradient centrifugation and plating for expansion (Macrin et al., 2017). A number of studies have shown their capacity to differentiate into mesodermal cell lineages (including myocytes, chondrocytes, osteoblasts and adipocytes), ectodermal cell lineages (such as neurons) and endodermal cell lineages (including hepatocytes). BM-MSCs also showed the capacity to be differentiated into airway epithelial cells, renal tubules, osteocytes and myocardial cells (Marolt Presen et al., 2019).

However, the frequency of MSCs in the bone marrow is very low (between 0.0001 and 0.01%) and decreases with age (Yang et al., 2018). In addition, their *ex vivo* expansion can only result in 30–50 population doublings, and long-term cell expansion may lead to chromosomal aberrations (Ahmadi and Rezaie, 2021). Additionally, bone marrow aspiration is a painful procedure that requires local anesthesia. Therefore, the use of BM has drawbacks, prompting the search for alternative sources of MSCs that are easily accessible, generally less invasive and contain larger amounts of MSCs.

#### 2.1.2 Adipose Tissue

Adipose tissue represents a very promising source for cell therapy purposes in terms of safety, collection and culture. Adipose MSCs are easy to obtain since isolation is performed under local anesthesia and presents little risk of morbidity (Seo et al., 2019). Adipose tissue therefore constitutes a source of MSCs in abundant quantities, and AT-MSCs have shown greater proliferation capacities than BM-MSCs. For the same amount of tissue aspirated, adipose tissue contains 550 × more MSCs than BM. The primary culture of adipose tissue-derived stem cells proceeds through the mincing and enzymatic digestion of subcutaneous adipose tissue followed by its culture and expansion in culture medium. These cells harbor several interesting characteristics and properties.

#### 2.1.3 Newborn Tissue

It has been reported that cord blood, placental amniotic membrane and fluid as well as the umbilical cord matrix (called Wharton’s jelly) contain MSCs (Liu et al., 2021). Perinatal tissues are of great interest due to their accessibility and ease of collection. The MSCs in these tissues are found at a high frequency and show an increased rate of proliferation and differentiation. Moreover, their use does not conflict with ethical issues raised by the use of embryonic stem cells (Aung et al., 2019). However, the cryopreservation step is essential, which can pose long-term storage problems (Peltzer et al., 2015).

#### 2.1.4 Peripheral Blood

MSCs have been shown to circulate at a low frequency in peripheral blood (Chen et al., 2019a). The origin of the presence of MSCs in this source is still under debate. This disparity could be linked to the diversity of isolation, culture and characterization methods used in different studies. The presence of MSCs in peripheral blood has been observed in patients with acute burns, suggesting the potential role of these cells in the regeneration of damaged tissue.

#### 2.1.5 Other Tissue Sources

Although MSCs from other sources, such as dental tissues, periodontal ligament, synovium, dermis, salivary gland, skin, and skeletal muscles, may share many biological characteristics, they also present differences regarding some properties (Mizukami and Swiech, 2018). Some of these differences, such as cell surface phenotype, transcriptome/proteome characteristics and immunotrophic activity, represent specific features of MSCs from a specific tissue source (Park et al., 2007), while others reflect the heterogeneity of MSC populations from different organs. Other differences may simply be attributed to the different isolation and culture protocols (Pelekanos et al., 2012). Other studies suggest that MSCs from different tissue sources retain an epigenetic memory of their original tissue. Thus, it has been shown that the expression profile of homeotic genes can vary from one source to another (Ackema and Charite, 2008). Additional studies have shown that the transcriptional expression of certain genes involved in the immunomodulatory function of MSCs could vary significantly depending on the cell source, even by changing the environmental conditions of culture (Cho et al., 2017; Fayyad-Kazan et al., 2017). Multiple comprehensive transcriptomic and proteomic analyses of human MSCs should help in identifying distinct populations of MSCs with distinct properties and specific clinical indications.

### 2.2 Phenotype: *In Vitro* Versus *In Vivo* Identity

Despite advances in MSC characterization and their wide use in regenerative medicine, their *in vivo* identity is still poorly understood. The isolation and purification of MSCs was achieved *via in vitro* phenotypic assays assessing the expression of specific cell markers (Table 4). Such analysis serves as an important quality control step that can save significant time and reduce experimental variability. The expression profile of several immunological molecules may influence the local immune-inflammatory response and, therefore, modulate the tissue healing process. By analyzing 27 relevant molecules, immunocomparative screening demonstrated that liver-derived stromal cells present a nonimmunogenic profile suitable to promote graft acceptance by the recipient (Merimi et al., 2021b).

**TABLE 4:** Markers differentially expressed by MSCs.

CD (cluster of differentiation)	MSC expression
CD3	−
CD9	+
CD10	+
CD11a	−
CD11b	−
CD13	+/−
CD14	−
CD15	+
CD16	−
CD19	−
CD29	+
CD31	+/−
CD34	+/−
CD35	−
CD36	+/−
CD38	−
CD40	+/−
CD44	+/−
CD45	+/−
CD49a	−
CD49b	+
CD49c	+
CD49d	+/−
CD49e	+
CD50	-
CD51	+
CD54	+/−
CD58	+/−
CD55	+
CD56	−
CD58	+
CD61	+/−
CD62e	−
CD62L	+/−
CD68	−
CD71	+
CD73	+
CD79	−
CD80	−
CD86	−
CD90	+
CD91	+
CD102	+/−
CD104	+/−
CD105	+
CD106	+/−
CD117	−
CD120a	+
CD120b	+
CD121a	+
CD124	+
CD133	−
CD134	−
CD140a	+
CD140b	+
CD144	+
CD146	+
CD164	+
CD166	+
CD200	+/−
CD252	−
CD221	+
CD271	+
CD274	+/−
SSEA-4	+
STRO-1	+
MSCA-1	+
HLA-ABC	+
HLA-DR	+/−
HLA-G	+/−

However, several artificial conditions during culture may introduce experimental artifacts and hide or impair the native identity of MSCs (Wilson et al., 2019a). Moreover, it is important that MSCs, after being cultured *in vitro*, retain all their receptors (to sense the tissue environment) and adhesion molecules (for migration, homing and cell-to-cell interaction) (Naji et al., 2019). MSC isolation methods, culture conditions and expansion may alter the expression profile of several markers. In addition to ISCT markers, other cell surface antigens, including nestin, CD29, CD44, CD49b, CD130, CD146, CD166, CD271, CD200, and αV/β5 integrin, have been reported. However, they are not specific to a tissue source of MSCs (da Silva Meirelles et al., 2008; Kuci et al., 2010). Other markers are expressed by MSCs, such as CD71, CD106, CD54, SUSD2, MSCA-1, and STRO-1 (Busser et al., 2015). However, accumulating evidence suggests that marker expression of MSCs is not stable in culture conditions, which renders MSC characterization based on their markers a challenge (Lv et al., 2014). Under the authority of the International Federation of Adipose Therapeutics (IFAT) and ISCT, a joint statement established minimal criteria for the definition of stromal cells from the adipose tissue-derived stromal vascular fraction (SVF) and culture-expanded adipose tissue-derived stromal/stem cells (Bourin et al., 2013). Evidence for CD34 as a common marker for diverse progenitors from adipose tissue, including MSCs, was thus reported. However, current literature has reported that the phenotype of MSCs can change during *ex vivo* expansion, which may represent alterations in the biological features of the MSC population involved in the response to environmental change. Today, no specific and unique marker can be used for isolating or identifying MSCs. Only markers of native mesenchymal stromal cells have been evaluated to enrich the population (Simmons and Torok-Storb, 1991). Positive selection for the CD140b (STRO-1) antigen increases the frequency of colony-forming unit fibroblasts (CFU-Fs) by 100-fold relative to the total cell population. Cells selected for the CD271 antigen have a better potential for proliferation and differentiation than the unselected population. Likewise, the CD200 and CD49a antigens allow significant enrichment of the population of mesenchymal stromal cells derived from the bone marrow by selecting the most multipotent cells (Rider et al., 2007; Delorme et al., 2008).

It has been shown that the MSC profile of cell surface antigens changes during cell culture. A previous study indicated that CD13, CD29, CD44, CD73, CD90, CD105, and CD106 in MSCs are downregulated during culture expansion compared to MSCs in the stromal fraction (Cao et al., 2020). As such, uncultured BM-MSCs isolated from both humans and mice do not express CD44 but express the surface protein (90% positive cells) after being plated in culture (Qian et al., 2012). In contrast to an increase in CD44, the expression of CD106 and CD271 on MSCs is decreased after culture (Jung et al., 2011). Typical markers of cultured MSCs, such as CD73 and CD105, appear to be expressed by the majority of freshly isolated MSCs and are maintained during culture. Currently, a critical marker, STRO-1, which has a high specificity for early passage bone marrow-derived MSCs, is not included in the ISCT criteria. This marker helps to identify, isolate, and characterize stromal progenitor cells. However, the expression of Stro-1 is lost from MSCs during *ex vivo* expansion, and it cannot be considered a valuable marker of MSCs. The selectivity of STRO-1 for cells that are not MSCs is not yet clear (Zhang et al., 2020). To identify relevant markers for the enrichment of MSCs from heterogeneous cultures, the expression of neuron-glial antigen 2 (NG2) and melanoma cell adhesion molecule (CD146) was investigated. The results showed that the expression of CD146 and NG2 was inversely correlated with doubling time during the serial passage of single-cell-derived human BM-MSC cultures. The fraction of MSCs with high expression of NG2 and low scatter properties is more clonogenic than the parental MSC culture from which it was derived (O’Connor, 2019). However, the expression of CD146 during *in vitro* culture showed discrepancies between studies, probably due to various factors, including donor variation, different culture conditions, immunostaining protocols and flow cytometry analysis. CD142 is another surface marker that may represent concern for the systemic administration of MSCs, as it is linked to thrombosis. BM-derived MSCs displayed less expression of CD142 than AT-MSCs. BM-derived MSCs are likely more suitable for intravenous delivery and decrease the risk of thrombosis (Christy et al., 2017; Le Blanc and Davies, 2018).

To date, the lack of specific markers to define MSCs poses an additional challenge in the field, and the use of more advanced molecular criteria has been proposed. Further, several research groups have attempted to develop novel markers, such as transcriptomic, epigenetic and proteomic markers (Wagner et al., 2016; Wiese et al., 2019; Wiese and Braid, 2020).

### 2.3 Tissue Repair Properties: Multilineage Potential Versus Paracrine Immunotrophic Actions

MSCs are able to migrate to inflamed areas and damaged sites where they promote tissue repair by different functions (Kim et al., 2021). The action of MSCs can be associated not only with a direct mechanism, through their differentiation and replacement of damaged cells, but also primarily with their paracrine properties that reduce the inflammatory response and stimulate (cell empowerment) the proliferation and differentiation of different local progenitor cells (Wang et al., 2014b; Dabrowska et al., 2020; Qiu et al., 2020; Chae et al., 2021). Hereafter, we present an overview of the current findings on the tissue repair properties of MSCs and their consequences for clinical application.

#### 2.3.1 Multilineage Potential

It was initially believed that MSCs mainly repair damaged tissues by cell-for-cell replacement driven by direct differentiation (Neshati et al., 2018; Smaida et al., 2020; Pan et al., 2021). Currently, there are no *in vivo* data demonstrating that MSCs differentiate into resident cells to repair injured tissue. Furthermore, permanent engraftment of MSCs into diseased tissues does not seem to occur. Therefore, the multipotency function of MSCs is likely an *in vitro* characteristic established to define MSCs a few years ago (Caplan, 2017). A number of culture protocols have been developed to induce MSC differentiation into several cell lineages in response to well-defined stimulation (Ullah et al., 2015) (Fitzsimmons et al., 2018). Although initially considered by Caplan to be stem cells, Sacchetti et al. (2007) revealed in 2007 that MSCs represent a rare and heterogeneous population of progenitors involved. The evidence supporting the *in vivo* differentiation of MSCs is relatively sparse and controversial compared to the abundance of data documenting *in vitro* multipotency. Most of the studies claiming *in vivo* differentiation, particularly beyond mesodermal-derived tissue types, have been methodologically flawed or have documented only extremely low frequency events. Relatively few studies have clearly demonstrated MSC engraftment with differentiation and functional incorporation into recipient tissues when subjected to critical review. This discrepancy between *in vitro* and *in vivo* evidence of MSC multipotency highlights the need for *in vivo* data supporting functional incorporation or tissue-specific gene expression of engrafted MSCs, where models of robust engraftment, incorporation and differentiation do not exist.

#### 2.3.2 Paracrine Immuno-Trophic Action

MSCs may act as immunomodulatory and trophic mediators in tissue regeneration and cell therapy. These actions imply interactions and interplay with local tissue cell progenitors as well as immune cells.

##### 2.3.2.1 The Trophic Process

Trophic function appears to have a critical role in mediating the beneficial effect of MSC therapy for degenerative and/or inflammatory diseases. In response to injury, homing receptors and chemokines are released, which subsequently activate MSCs. Activated MSCs are then mobilized into the peripheral blood circulation, where an adhesion step is achieved by the specific interaction between chemokines and homing receptors such as stromal cell-derived factor (SDF-1), CXC chemokine receptor (CXCR) 4, hepatocyte growth factor (HGF), c-Met, hyaluronic acid (HA), CD44, monocyte chemoattractant proteins (MCPs) and C-C chemokine receptor type 2—CCR2/CD19. The transendothelial migration of MSCs to the local site of injury occurs via the degradation of extracellular matrix (ECM) by matrix metalloproteinases (MMPs) (Lin et al., 2017). Through a plethora of molecules (including HGF, IGF, VEGF, TGF-β1, and FGF-2), MSCs may regulate tissue homeostasis within the stromal niches by supporting the maintenance, expansion and/or differentiation of local resident cells (Park et al., 2018). MSCs can produce large amounts of growth factors, which subsequently stimulate endothelial cells, fibroblasts and, most importantly, tissue progenitor cells or stem cells *in situ*. The concerted action of these factors and cells facilitates tissue repair through angiogenesis, remodeling of the extracellular matrix (ECM) and the differentiation of tissue progenitor cells (Wang et al., 2014b).

##### 2.3.2.2 The Immunomodulatory Process

MSCs are able to suppress the activity of the immune system and help resolve inflammation. However, MSCs are also able to stimulate the response of the immune system. This ability has therefore led some authors to hypothesize that MSCs could adopt, depending on the context, a pro- or anti-inflammatory phenotype (Bernardo and Fibbe, 2013; Betancourt, 2013).

MSCs modulate both inflammatory and immune responses by regulating innate and adaptive immunity that favor tissue repair (Chen et al., 2019b; Song et al., 2020b) Their effects are not HLA (human leukocyte antigen)-restricted. MSCs act on all effectors of innate and adaptive immunity and alter cell proliferation and other functions of immune cells. In addition, MSCs can inhibit the proliferation, cytotoxicity and production of IFN-γ in T lymphocytes and NK cells (Prigione et al., 2009). Blocking G0/G1 phases of the cell cycle, inducing apoptotic pathways and impairing the T cell subset ratio and inhibiting dendritic cells are among the mechanisms to inhibit lymphocyte proliferation (You et al., 2019). MSCs may also promote the polarization of macrophages from a proinflammatory phenotype to an anti-inflammatory phenotype, promoting tissue regeneration (Sica and Mantovani, 2012). MSCs also inhibit B lymphocyte proliferation and alter their differentiation into plasma cells. In addition, MSCs may induce the differentiation of regulatory T lymphocytes (Saeedi et al., 2019).

Several mechanisms have been proposed to explain the immunomodulation carried out by MSCs, among which are (Najar et al., 2016):

- Indoleamine 2,3-dioxygenase (IDO), which catabolizes tryptophan to kynurenine. IDO is crucial for the inhibition of effector T lymphocyte proliferation by human MSCs and acts by depleting the medium of an essential amino acid, tryptophan, and by producing kynurenine, which is toxic to T lymphocytes.
- Prostaglandin E2 (PGE2), in combination with IDO, participates in the inhibition of NK cell proliferation.
- TNF-α stimulates “gene/protein 6” (TSG-6), which acts by a negative feedback control on macrophages by reducing their synthesis of proinflammatory factors, which in particular decreases the recruitment of polymorphonuclear neutrophils within the damaged tissues.
- HLA-G5, which is thought to be responsible for the production of regulatory T lymphocytes;—“Transforming growth factor”-β (TGF-β), galectins, adenosine and FAS pathways, “programmed cell death protein 1” (PD-1), IL-1RA, IL-10 and Notch, but none of these mechanisms alone summarizes the immunomodulatory activity of MSCs.

Among the proven elements, MSCs are not constitutively immunosuppressive but acquire these properties after stimulation by inflammatory signals from the microenvironment, such as the inflammatory cytokines IFN-γ or TNF-α. MSCs are able to secrete a set of proinflammatory molecules, including IL-6, IL-8, GM-CSF (granulocyte-macrophage colony-stimulating factor) and MIF (macrophage migration inhibitory factor), thus promoting the recruitment and survival of neutrophils. Under the effect of these same signals, it has been shown that MSCs were able to block the synthesis of IL-10 from B lymphocytes, thus promoting a proinflammatory response. Mechanistically, MSCs likely contribute to immunomodulation through cell-to-cell contact or paracrine effects (Burnham et al., 2020). The secretome of MSCs comprises various cytokines and regulatory factors (e.g., TSG-6, TGF-b, hepatocyte growth factor, IFN-γ, prostaglandin E2, PGE2 and IDO pathways), insulin-like growth factor binding proteins, heme oxygenase-1 (HO), human histocompatibility antigen-G5 (HLA-G5), chemokine (C-C motif) ligand 2 (CCL2), IL-10, galectin-1 and galectin-3 (Madrigal et al., 2014; Burnham et al., 2020). The inflammatory context could also lead MSCs to synthesize a set of chemokines, such as CCL2, CCL3 or CCL12, helping the recruitment of macrophages, or like CXCL9, CXCL10 or CXCL11, promoting the chemotaxis of T lymphocytes (Andrzejewska et al., 2019b; O’Connor, 2019). These paracrine pathways may explain the therapeutic effect of MSCs despite their low engraftment, homing and survival after transplantation (Cruz et al., 2017).

##### 2.3.2.3 Extracellular Vesicles

Extracellular vesicles (EVs) are membrane-enclosed heterogeneous structures including exosomes, microvesicles, ectosomes, microparticular membrane particles, exosome-like vesicles and apoptotic bodies that are released into the extracellular space. However, the defining parameters for each of these different classes are not definitive, and the use of the terms exosomes, microvesicles, and microparticles is often ambiguous and not rigorously qualified. These structures have been shown to participate in a wide variety of biological processes and are currently under intense investigation in many different fields of biomedicine (Buzas et al., 2018). EVs may be secreted by multiple types of cells and have been demonstrated to mediate intercellular communication in both physiological and pathological conditions. Generally, EVs can be formed by either inward budding of endolysosomal vesicles followed by exocytosis (e.g., exosomes) or shedding from the plasma membrane (e.g., microvesicles) (Klyachko et al., 2020). Due to their ability to carry key molecules, EVs affect the physiological and pathological functions of recipient cells. Generally, EVs carry a cargo of proteins and nucleic acids that reflect their cell of origin. They represent a sophisticated intercellular communication system and potential healing agents or delivery vehicles of therapeutic agents. Studies have confirmed that a major portion of the beneficial proprieties of MSCs arises from their paracrine activities (Varderidou-Minasian and Lorenowicz, 2020). EVs derived from MSCs may deliver a variety of molecules to the surrounding cells, leading to functional changes in the recipient cells (Hong et al., 2019). The regenerative and immunomodulatory capacity of MSC-derived EVs has been evaluated in several animal disease models, including kidney and liver injury, lung disease, cartilage repair, hind limb ischemia, ischemic brain injury, and spinal cord injury (Harrell et al., 2019; Hu et al., 2019; Liau et al., 2019). MSC-derived EVs represent a potential cell-free therapeutic option, as they are a major key for crosstalk communication between cells.

### 2.4 Microenvironmental Cues Influencing the Function of Mesenchymal Stem/Stromal Cells

MSCs are considered responsive cells because they are able to sense the tissue environment and adapt their features accordingly. Such capacity to adjust their properties is linked to their functional plasticity. Such plasticity allows MSCs to actively respond to local tissue challenges and therefore display the appropriate therapeutic response (Wang et al., 2014b). Several *in vitro* strategies, including the use of relevant factors/conditions, have been reported to likely modulate the properties of MSCs (Wilson et al., 2019b).

These approaches require appropriate controls before translation for clinical applications because of the risk of immunogenicity, tumorigenicity, epigenetic modifications, loss of viability and efficiency. The production of MSCs using these approaches should be accomplished according to GMP.

#### 2.4.1 Oxygen Saturation

The oxygen (O_2_) content under normoxic conditions is 21%, which is of course higher than the concentrations found in the organs of the body (1–10%). For this, the culture of MSCs under hypoxic conditions with an O_2_ percentage ranging from 1 to 10% clearly improves the proliferation of MSCs, their survival and the conservation of their multipotent character by keeping them in an undifferentiated state (Elabd et al., 2018). Some studies have also shown an improvement in the paracrine activity of MSCs through increased production of IL-6 and growth factors VEGF, HGF and bFGF (basic fibroblast growth factor). The effect of the hypoxic environment is mainly due to the induction of the transcription factor HIF-1 (hypoxia-inducible Factor 1), which in turn can interfere in different signaling pathways and induce the expression of the target genes involved in angiogenesis, proliferation and metabolism of MSCs (Luo et al., 2019).

#### 2.4.2 Three-Dimensional Culture of Mesenchymal Stem/Stromal Cells

Three-dimensional (3D) culture is another strategy to enhance the potential of MSCs. Culturing MSCs in spheroids creates a hypoxic environment that strengthens their survival and proliferation. In addition, the anti-inflammatory, antifibrotic and proangiogenic activities of these MSCs are improved following an increase in the expression of the immunoregulatory factors TSG6 (TNFα-stimulated gene-6), PGE2 (prostaglandin E2) and IL-6 as well as trophic factors STC-1 (stanniocalcin 1), CXCR4, angiogenin and VEGF (Tsai et al., 2015). The use of specific biomaterials has demonstrated significant improvement in MSC therapy. Antonini et al. (2016) showed that the use of polyethylene terephthalate nanogratings improved the osteogenic differentiation of MSCs.

#### 2.4.3 *In Vitro* Toll-Like Receptors Triggering

The Toll-like receptor (TLR) signaling pathway plays critical roles in the inflammatory response as well as in the regulation of tissue injury and wound healing processes. Depending on their origin and culture condition, MSCs can differentially express several patterns of TLRs. The engagement of these TLRs by their respective ligands results in different biological and immunomodulatory responses by MSCs (Tomchuck et al., 2008). MSCs can adopt pro-inflammatory or anti-inflammatory functions of the MSC1/MSC2 type depending on TLR engagement (Waterman et al., 2010). It was shown that the binding of LPS with TLR4 induces MSC differentiation into a proinflammatory phenotype with high expression of IL-6 and IL-8 and induction of T lymphocyte proliferation cocultured with MSCs (Waterman et al., 2010). On the other hand, the binding of poly (I:C) (polyinosinic-polycytidylic acid) with TLR3 polarizes MSCs toward an anti-inflammatory phenotype with high expression of IL-4, IDO and PGE2 and retains their immunosuppressive effect on T lymphocytes (Waterman et al., 2010). However, contrary to these results, (Liotta et al., 2008) showed an inhibition of the immunosuppressive effect of MSCs independent of TLR3 or TLR4 engagement by the inhibition of the Notch signaling pathway induced by Jagged-1. These contradictory results can be explained by differences in the culture conditions between the distinct studies, the concentrations of ligands used and the duration of treatment. TLRs are therefore important regulators of MSC functions and deserve more in-depth and standardized studies to better understand the influence of their ligands on the potential of MSCs.

#### 2.4.4 *In Vitro* Inflammatory Licensing

Within the injured tissue, a plethora of inflammatory mediators and cytokines are released that may influence the function of MSCs. The immunomodulatory functions of MSCs are regulated by the complexity and intensity of the inflammatory environment. MSCs express several receptors on their surface for inflammatory mediators, such as IFN-γ, TNF-α, IL-1 and IL-6, which makes them capable of perceiving and reacting significantly to inflammatory stimuli. Accordingly, the immunosuppressive properties of MSCs were increased by IFN-γ in *in vitro* and *in vivo* models (Kim et al., 2018). IFN-γ licensing induced IDO expression in MSCs via the JAK/STAT1 signaling pathway. Moreover, it has been shown that MSCs have substantial and beneficial anti-inflammatory effects in the mouse model of GvHD when the latter were injected during the inflammatory peak. This effect was lost when anti-IFN-γ antibody was injected in parallel to WT-MSCs (wild-type MSCs) or when IFNγR1^−/−^ MSCs were used (Ren et al., 2008). In the presence of IFN-γ and TNF-α, the expression of the chemokines CXCL9, CXCL10, RANTES (regulated upon activation, normal T cell expressed and presumably secreted) and CCL3 was considerably induced and involved in the recruitment of immune cells (specifically lymphocytes) to the surroundings of MSCs. They also induced the expression of ICAM-1 (intercellular adhesion molecule 1) and VCAM-1 (vascular cell adhesion molecule 1) molecules that facilitate cell adhesion, and ultimately, they stimulated the production of large amounts of IDO, iNOS and PGE2 involved directly in the immunomodulatory effect of MSCs (Ren et al., 2008; Li et al., 2012; Kim and Cho, 2016). On the other hand, during chronic or controlled inflammation, where the concentrations of IFN-γ and TNF-α are suboptimal, the latter induced the expression of chemokines but was insufficient to induce the production of the soluble mediators IDO and NO in large quantities, which would have a countereffect: the cells will be recruited near the MSCs without being inhibited, and in this case, the inflammatory process is aggravated (Kim and Cho, 2016).

A recent profiling highlighted that following a combination of inflammatory and proliferative signals, the sensitivity and responsive capacity of AT-MSCs were significantly modified (Merimi et al., 2021a). In particular, inflammation leads to an upregulation of IL-6, IL-8, IL-1β, TNF-α and CCL5 cytokine expression. Inflammation and cell passaging increased the expression of HGF, IDO1, PTGS1, PTGS2 and TGFβ. The expression of the TLR pattern was differentially modulated, with TLR 1, 2, 3, 4, 9 and 10 being increased, whereas TLR 5 and 6 were downregulated. Such observations are encouraging and have to be developed as preconditioning strategies to strengthen MSC function and proprieties (Muller et al., 2018).

#### 2.4.5 Orthobiologics

MSCs hold promise for tissue healing, but some criticisms hamper their clinical application, including the need to avoid xenogeneic compound (e.g., animal serum) contamination during *ex vivo* cell expansion and scarce survival after transplantation. Orthobiologics are biological substances used to improve tissue healing and include platelet-rich plasma (PRP) and platelet lysate (PL) (Kruel et al., 2021). Many studies have demonstrated the ability of PRP, a source of many biologically active molecules, particularly growth factors, to positively influence MSC proliferation, survival and functionality, as well as its antifibrotic potential. Previous results suggested that PRP is able to positively affect BM-MSC viability, survival and proliferation, suggesting that it could represent a good serum substitute during *in vitro* cell expansion and could be beneficial toward transplanted cells *in vivo* (Sassoli et al., 2018). In parallel, the proliferation, cell cycle, and migration of umbilical cord-derived MSCs (hUC-MSCs) was significantly promoted in the presence of PL by upregulating relevant genes/proteins (PDGF-AA, IGF-1, TGF-β, EGF and FGF) and activating beclin1-dependent autophagy via the AMPK/mTOR signaling pathway (Yan et al., 2020).

Additionally, PRP may provide a suitable microenvironment that potentiates the enhancement of the functionality of MSCs. In this way, PRP and AT-MSC combined therapy significantly accelerated the healing of diabetic wounds induced experimentally in rats by modulating the Notch pathway, promoting angiogenesis and the proliferation of epidermal stem cells (EPSCs) (Ebrahim et al., 2021).

A study indicated that PRP improved the efficacy of engrafted MSCs to replace lost skin in mice by accelerating the wound healing processes, ameliorating the elasticity of the newly regenerated skin and stimulating their proangiogenic potential through enhanced secretion of soluble factors such as VEGF and SDF-1. These effects were also accompanied by an alteration of MSC energetic metabolism, including the oxygen consumption rate and mitochondrial ATP production (Hersant et al., 2019). Accordingly, there is a need to identify appropriate (regarding safety and efficiency) growth factors acting as preconditioning agents that may improve the cell survival, proliferation and function of MSCs within the host tissue microenvironment.

### 2.5 Engineering Mesenchymal Stem/Stromal Cells

Genetic engineering has emerged as another challenging yet promising approach to improve the therapeutic properties of MSCs. In fact, MSCs can be genetically engineered to overexpress certain desired elements and soluble factors, such as growth factors, cytokines, chemokines, transcription factors, enzymes and microRNAs (Fricova et al., 2020; Miceli et al., 2021). Distinct strategies have been applied to induce genetic modifications to further enhance the therapeutic potential of MSCs by improving various cellular properties, such as survival, homing and immunomodulatory effects. Several studies have demonstrated the use of genetic engineering (Baldari et al., 2017). Several studies using engineered MSCs have investigated the role of pancreatic duodenal homeobox-1 (PDX-1) and VEGF to produce functional insulin-producing cells as cellular therapy for diabetes; *β*-glucuronidase (GUSB) gene to improve genetic enzyme deficiency mucopolysaccharidosis type VII (MPSVII); IFN-α and INF-β in cancer therapy; Bcl-xL to stimulate angiogenesis; Bcl-2, heme-oxygenase-1 and Akt1 to improve the cell survival helping heart tissue repair in myocardial infarction; BMPs, to induce osteogenic differentiation; Neurogenin1 (Ngn1) to induce neuronal differentiation and lipocalin2 (Lcn2) to restore the renewal potential of MSCs (Noronha et al., 2019; Saeedi et al., 2019). Viral vector–based genetic engineering typically has more efficient and durable gene expression but has some safety concerns because genes are integrated into the target cell genome. Nonviral vectors are safer, but the transfection efficiency is typically lower and gene expression is less durable. MSCs can also be engineered with drug-loaded particles. These particles are intracellularly loaded into MSCs to sustain their immunosuppressive profile for an extended period, regardless of the source of MSCs, but particle preparation can increase the cost and complexity when compared to the use of free small molecules. Oncolytic virus (OV) engineering has also been used to engineer MSCs. MSCs function by shielding viruses to avoid immunogenicity and by releasing the virus in tumor tissue to kill tumor cells. One limitation is that regular OVs have only moderate infectivity, although this can be overcome by using certain viral variants with higher infectious capacity (Levy et al., 2020).

### 2.6 Aging and Senescence

Although data on the functionality of MSCs isolated from aged subjects versus young individuals are still under debate in the literature, some consensual evidence appears. With increasing donor age, MSCs from bone marrow are reported to show a decrease in proliferative and clonogenic/self-renewal capacities, characterized by a number of CFU-Fs, but no phenotypic change is correlated with age (Charif et al., 2017). On the other hand, other studies reported the absence of substantial differences between cells from adult and elderly cohorts; therefore, aging rather than *in vivo* donor aging influences MSC characteristics. Indeed, (Andrzejewska et al., 2019a) compared MSCs from cohorts of young and old donors by analyzing their phenotypic and functional performance, using multiple assays typically employed as minimal criteria for defining MSCs. They found that MSCs from both cohorts met the standard criteria for MSCs, exhibiting similar morphology, growth kinetics, gene expression profiles, proangiogenic and immunosuppressive potential and the capacity to differentiate toward adipogenic, chondrogenic and osteogenic lineages.

The number of population doublings required for obtaining sufficient numbers of MSCs for therapy would be dependent on the initial number of viable MSCs. Therefore, attaining sufficient numbers could be subject to a large number of population doublings with the attendant possibility of stemness attenuation and cellular senescence (Liau et al., 2019). It has been reported that prolonged MSC expansion is accompanied by phenotypical and morphological changes, such as enlarged and irregular cell shapes and shortened telomere lengths, as well as gene, miRNA and protein expression alterations in cells, which ultimately lead to a state of senescence. Cellular senescence is generally defined as an arrest of cell proliferation. Replicative senescence refers to irreversible growth arrest of human diploid cell strains after extensive serial passaging in culture (Zhai et al., 2019). The presence of senescent cells in therapeutic MSC batches is undesirable, as it reduces their viability, differentiation potential and trophic capabilities. It is well documented that human MSCs (hMSCs) lose their differentiation potential after prolonged culture expansion *in vitro* and that cells from late, presenescent passages may not be able to differentiate at all. Additionally, their presence in MSC culture negatively influenced immunomodulatory and homing properties (Turinetto et al., 2016; Robb et al., 2019). Additionally, senescent cells acquire a senescence-activated secretory phenotype, which may not only induce apoptosis in neighboring host cells following MSC transplantation but also trigger an age-related disease phenotype such as osteoarthritis. Current methods for MSC senescence analysis in culture have been developed and were comprehensibly described previously (Zhai et al., 2019).

## 3 The Clinical Challenges

**FIGURE 3:**
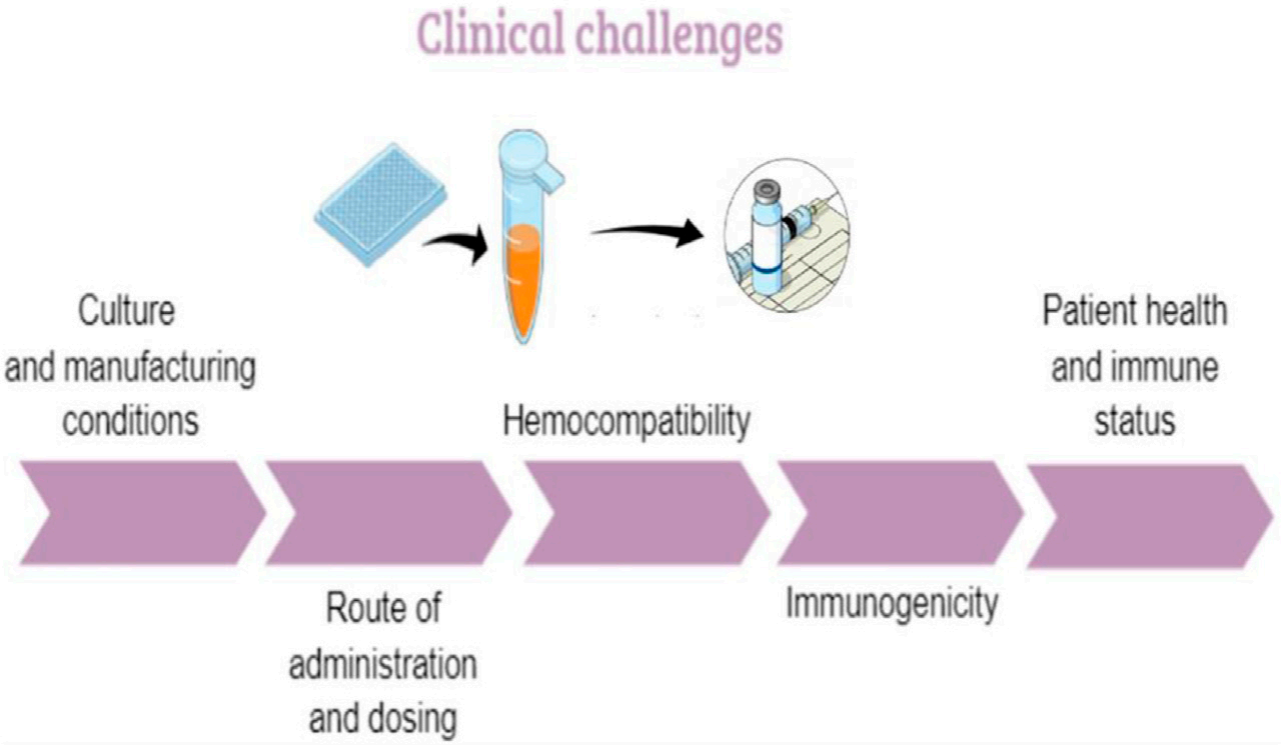
The clinical challenges linked to MSCs.

Several clinical challenges may influence the therapeutic use of MSCs and should be well identified and characterized (Figure 3).

### 3.1 Culturing and Manufacturing Conditions

As the frequency of MSCs is low after isolation, there is a need to expand the cells *ex vivo* to a high number before their use. Thus, the culture and manufacturing conditions may influence the properties of MSCs and should be well identified. On a large scale, and in accordance with good manufacturing practices (GMPs), MSCs are expanded with bioreactors. A bioreactor is a culture system where all conditions, including pH, temperature, and oxygen level, can be managed and controlled for proper cell expansion. Different types of bioreactors are used in MSC expansion, such as stirred tank bioreactors, rocking bioreactors, hollow fiber bioreactors and fixed-bed bioreactors (Mizukami and Swiech, 2018). It should be noted that these different culturing protocols and systems directly affect the therapeutic potential of MSCs; thus, each trial follows restricted rules to obtain the desired final product.

Additionally, the identification of optimal culture conditions is a prerequisite for MSC clinical applications. Animal-derived growth supplements, such as fetal bovine serum (FBS), have been predominantly used for MSC expansion. However, utilization of animal-derived products bears critical limitations and safety concerns. In particular, the risk of contamination and transmission of infectious agents, the potential to activate xenogeneic immune responses and animal welfare should not be neglected. Moreover, the exact composition of FBS remains unclear, and there are often significant variations between lots. Hence, it is necessary to determine suitable alternatives to animal serum that comply with all the relevant clinical requirements and that provide the appropriate quantity of high-quality cells while preserving the required properties. Alternative animal product-free formulations, including human AB serum (HABS), human platelet lysate (HPL) and chemically defined media (CDM), have been developed (Oikonomopoulos et al., 2015; Yin et al., 2019). Despite their batch-to-batch variability, these alternatives resolve most of the basic problems associated with the application of FBS. Although they represent promising supplements due to their native and human origin, further detailed analysis and studies will be required, and guidelines will have to be set to fully guarantee the safety and efficiency of these alternatives. The different manipulation and storage procedures (freeze-thawed or freshly harvested) may also affect the quality of the product. Most of the allogenic MSCs used in clinical trials are cryopreserved. However, studies have shown that the cryopreservation of MSCs reduces their immunomodulatory and blood regulating properties (Moll et al., 2014a; Moll et al., 2016). Further research is imperative for the optimization of culturing and manufacturing conditions to ensure a better cellular “fitness” of MSCs.

### 3.2 Route of Application and Dosing

The route of MSC administration is highly dependent on the desired curative ability. Systemic (intravenous IV, intra-arterial IA, inhalation) and local (topical, direct tissue injection, intramuscular, *trans*-epi, *trans*-endocardial, intra-articular) delivery are common routes for the administration of MSCs (Caplan et al., 2019). In addition to the route of administration, the effective and accurate number of MSCs, the number of doses (single or repeated doses) and the interval of time between each dose are among the challenges influencing the safety and efficacy of the therapy (Galipeau and Sensebe, 2018; Kabat et al., 2020).

### 3.3 Hemocompatibility

MSCs are ABO neutral, and research has demonstrated that they do not inherently express ABO blood group antigens. However, the use of human AB plasma (ABP) while working with MSCs led to an adsorption of ABO antigens proportional to antigen concentration in the serum and adsorption time. Thus, particularly when treating immunocompetent patients or patients with blood type O, it is recommended to wash and infuse MSCs with nonimmunogenic human serum albumin (Moll et al., 2014b; Olsen et al., 2018). In some cases, MSCs initiate instant blood-mediated inflammatory reactions (IBMIRs). This later significantly causes the failure of allogenic graft survival and function. MSC hemocompatibility is mainly determined by procoagulant tissue factor (TF), which is highly correlated with the initiation of IBMIR. BM-MSCs show a lower expression of TF than MSCs from other sources (adipose tissue, perinatal tissue). Thus, MSCs are largely used in clinical trials with intravenous administration to minimize the rate of IBMIR and prolong engraftment survival. Nevertheless, many studies have investigated the effect of many conditions (such as culture media, freeze-thawing and cell expansion) on the ability of MSCs to trigger IBMIR (Moll et al., 2019). However, for clinical applications, it is suggested to add anticoagulant factors with MSC transplantation (Oeller et al., 2018).

### 3.4 Complement

Understanding the behavior of MSCs after infusion is still the focus of many studies. Culture-expanded human MSCs may elicit an innate immune attack, termed IBMIR. This reaction is characterized by the activation of the complement cascades. This deleterious reaction can compromise the survival, engraftment, and function of these therapeutic cells (Moll et al., 2012). MSCs have a short lifespan after *in vivo* administration and rapidly disappear from tissues. Such an observation does not rule out a beneficial effect of MSCs. It has been reported that the phagocytosis of MSCs may induce the generation of regulatory monocytes. It is also possible that a small proportion of MSCs escape this clean-up process and are responsible for the therapeutic effects (Eggenhofer et al., 2014). In line with this, some circulating MSCs, present at a very low level in healthy individuals, may greatly increase under specific conditions. After being mobilized, these local MSCs are recruited to the site of injury where they participate in the healing process (Xu and Li, 2014). Another hypothesis supports that the very rare presence of MSCs is likely linked to biophysical microdamage rather than the fact that specific molecular cues to a circulatory pool of MSCs are capable of repairing remote organs or tissues (Churchman et al., 2020). Several groups have revealed that MSCs, after infusion, activate complement by unknown mechanisms, leading to their damage and disappearance. The complement system, a part of the innate immune response, helps to remove microbes and damaged cells in parallel to promoting inflammation. Despite its importance, there are few studies investigating the interaction between complement and MSCs. A major role of the complement system during the interaction of MSCs with immune cells as well as in modulating their therapeutic activity was previously described (Moll et al., 2011). The complement-activating properties of MSCs were correlated with their potency to inhibit peripheral blood mononuclear cell proliferation *in vitro*. It was suggested that MSCs could be phagocytosed and removed by monocytes, which participate in their immunomodulatory properties (de Witte et al., 2018). It is proposed that complement opsonization induces phagocytosis of MSCs by monocytes after their intravenous infusion. Indeed, despite the expression of complement inhibitors, including CD46, CD55 and CD59, MSCs are injured after complement binding. Such phagocytosis may induce anti-inflammatory and pro-regenerative M2 monocyte polarization that could explain the therapeutic functions of MSCs (Gavin et al., 2019b). In contrast, some results indicated that complement activation is integrally involved in recognizing and injuring MSCs after their infusion (Li and Lin, 2012). The inhibition of complement activation could be a novel strategy to improve the efficiency of MSC-based therapies. The cell-surface engineering of MSCs with heparin has improved the viability and functions of MSCs after infusion by directly inhibiting complement and by recruiting Factor H, another potent complement inhibitor (Li et al., 2016). As an alternative to other sources of MSCs, placenta-derived decidual stromal cells (DSCs) were shown to be therapeutically efficient. Although complement activation was observed, this effect was particularly decreased when DSCs were supplemented with low-dose heparin (Sadeghi et al., 2019). A previous study found that incubation with autologous serum damaged BM-MSCs, probably following the formation of the complement membrane attack complex (MAC) induced by complement activation. Membrane complement regulatory proteins (mCRPs) can inhibit the activation of complement and thus prevent tissues from being damaged. It was thus suggested that the clinical use of mCRPs during the transplantation of MSCs can decrease the cytotoxicity induced by complement activation and therefore guarantee the survival and function of these therapeutic cells (Xiao et al., 2017). Deep investigation of the interplay between MSCs and complement activation might be a straightforward and effective step for improving the outcome of current MSC-based therapies.

### 3.5 Immunogenicity

Because of the lack of consistent assays to measure their specific immunogenicity, MSCs have long been reported to be hypoimmunogenic or “immune privileged.” MSCs should be considered immunoevasive cells with low immunogenicity. Depending on the conditions, MSCs do not express HLA class II, and their expression for HLA class I is low, preventing the activation of allorecognition pathways. The expression of HLA, CD40, CD80 and CD86 costimulatory molecules can be influenced by the inflammatory status within the surroundings of MSCs (Dominici et al., 2006). Indeed, the generation of antibodies against MSCs and the possible immune rejection in an allogeneic donor suggest that these cells may not be immune privileged (Ankrum et al., 2014). They can be recognized by the immune system and predisposed to be destroyed by cytotoxic immune cells such as natural killer (NK) cells or cytotoxic T lymphocytes (CTLs)In some “off-the-shelf” allogenic cases, cellular and humoral immune responses were observed. Allogenic BM-MSC injection with MHC mismatch in animal models initiates an immune reaction and therefore leads to transplant rejection. Research studies have explained that the expression of MHC/HLA is altered due to several factors, such as culturing conditions and epigenetic modification (Kot et al., 2019). Moreover, MSC differentiation leads to the upregulation of immunogenic molecules on the cell surface and thus an increase in MSC immunogenicity (Lohan et al., 2017). In addition, a high number of passages for MSCs increases inflammatory reactions after systemic administration. Within immunocompetent mice, allogeneic MSCs provoked an immunogenic response, with the infiltration of inflammatory cells at the transplant site and full graft rejection. Allogeneic islets cotransplanted with preactivated MSCs prolonged graft survival by approximately 6 days compared with islets alone. Such an observation corroborates the hypothesis that allogeneic MSCs are not immune-privileged and that after playing their therapeutic role, they are rejected (Oliveira et al., 2017). To resolve the immunogenicity challenges, two features must be investigated. First, modern assays to appropriately identify and measure immune responses to MHC-mismatched MSCs should be developed (Berglund et al., 2017). Second, new engineering approaches should be applied to overcome the rejection of allo-MSCs, avoid the generation of alloreactive antibodies in parallel to prolong their *in vivo* survival and engraftment and enhance their immunoregulatory paracrine activity.

### 3.6 Patient Health and Immune Status

Although the biological characteristics of the injected donor cells are inarguably one of the most important factors that determine the efficacy of MSCs, the recipient environment where immunomodulation is supposed to take place should not be neglected. For example, age, skin involvement, lower acute GvHD grade, and the number of infusions are the main prognostic factors affecting the efficacy of MSC therapy for steroid-refractory acute GvHD (Chen et al., 2015). The recipient immune environment can influence the therapeutic outcome following the use of MSCs. As shown by Gavin et al. (2019a), Gavin et al. (2019b) a proinflammatory immune profile within the gut at the point of MSC treatment may impede their therapeutic potential for GvHD. The recipient immune environment can also vary according to the age of the patient. Physiological aging is accompanied by a decline in immune system function. Age-related changes from infants through adults revealed progressive declines in the percentage of total lymphocytes and absolute numbers of T and B cells. The proinflammatory cytokines TNF-α and IL-6 were higher in elderly people than in adults (Valiathan et al., 2016). It is now generally recognized that the immunomodulatory properties of MSCs are not constitutive but are induced by various mediators present in the inflammatory environment. Different inflammatory stimuli are able to polarize MSCs with distinct phenotypes and functions. Inflammatory status changes throughout the course of an immune response and is affected by time, activators of the immune system and many other factors. Therefore, it is likely that the types and amounts of inflammatory cytokines present in the stromal niche will dictate the migration and function of MSCs (Wang et al., 2014b). Thus, adipose-derived MSCs significantly reduced the severity of experimental autoimmune encephalomyelitis (Ebrahim et al., 2021) by suppressing the autoimmune response in early phases of disease and not during disease remission (Constantin et al., 2009). Compared with adults, children generally showed a trend toward better complete responses (Introna et al., 2014; Chen et al., 2015). A multicenter nonrandomized phase II study addressing the infusion of MSCs in patients with severe steroid refractory showed that children responded consistently better than adults, with more complete remissions and less progressive disease (Le Blanc et al., 2008). In addition, multiple infusions of MSCs were more effective for children with steroid-refractory acute disease, especially when employed early in the disease course (Ball et al., 2013). MSCs from pooled bone marrow mononuclear cells of several healthy third-party donors were more effective in the treatment of severe acute GvHD (Kuci et al., 2016). As mentioned previously, preparation of the patient’s body with anticoagulants is also necessary in some cases to prevent the initiation of IBMIR and hopefully lead to a better outcome (Moll et al., 2019). In another report, it was assumed that a part of the therapeutic effect of MSCs was mediated by host/patient phagocytic cells. The latter help to remove MSCs administered to the patient and thus modulate MSC activity (Hoogduijn and Lombardo, 2019). Such observations indicate the need to further establish the immune cell profile of patients who may segregate responders from nonresponders to MSC therapy. It is recommended to explore and monitor the inflammatory and immunological status of patients at the time MSCs are infused to help optimize MSC-based therapy. Moreover, discussions about the relevance of preconditioning MSCs before transplantation and the identification of biomarkers to predict patient responsiveness to MSC therapy are ongoing.

### 3.7 Clinical Optimization

**FIGURE 4:**
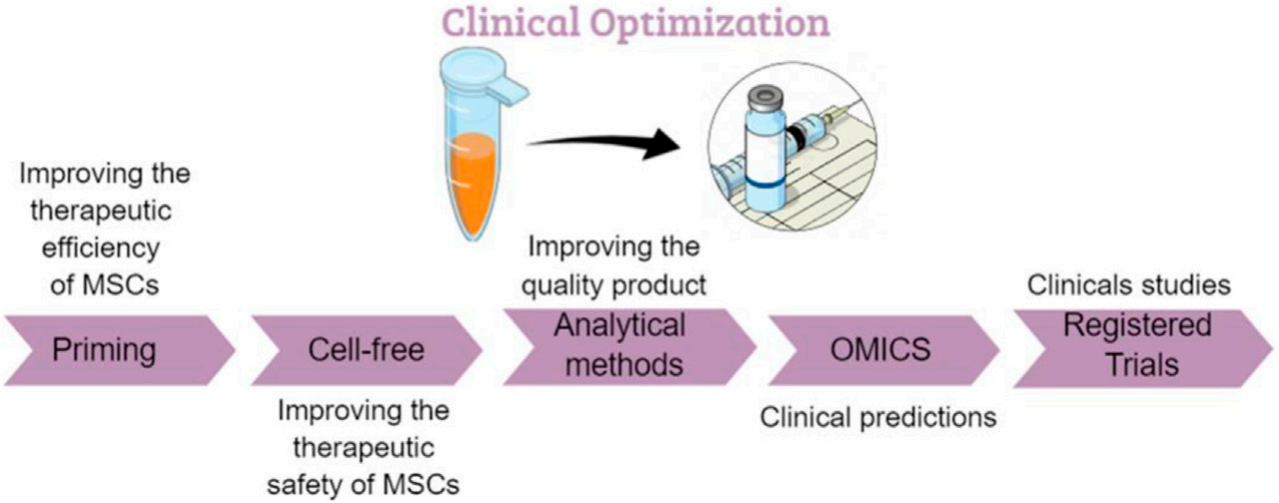
The clinical optimization of MSC therapy.

The clinical optimization of MSCs is required to achieve safe and efficient therapeutic indications (Figure 4).

#### 3.7.1 Cell-Free Therapeutic

As previously discussed, several benefits and advantages are linked to the use of EVs isolated from MSC-conditioned media as a cell-free therapy. EVs have many advantages over MSCs; they are easy to dose, prepare, store and administer at the time of choice, cost less, are small and have no risk of vascular obstruction (Phinney and Pittenger, 2017). Based on these data, researchers are leaning toward their use as potential therapies for several diseases. EVs are likely heterogeneous and differ depending on the type of MSCs from which they are derived. Thus, well-defined and characterized EVs are recommended. Moreover, their metabolomic and lipidomic profiles have not yet been well characterized. Other limitations of EV isolation and purification involve the procedure itself, which includes variability in the quality of EV preparations, the yield of EVs, and the potential for non-EV contaminants in the preparation. Likewise, the production and packaging methods for the vesicles produced by MSCs are currently being validated.

In this context, cell-free therapies involving the secretome of MSCs have, in theory, lower safety risks than cellular products. Indeed, these therapies cannot replicate as cells, but this estimated safety risk cannot ignore the risk of influencing tumorigenesis. In line with these findings, several studies have reported that EVs shed by cancer stem cells (CSCs) may significantly contribute to tumor progression. CSC-derived EVs are involved in tumor resistance, metastasis, angiogenesis, maintenance of the stemness phenotype and tumor immunosuppression microenvironment (Su et al., 2021). As stated by the Cell Products Working Party and the Committee for Advanced Therapies of the ISCT, the risk of potential tumorigenicity related to MSC-based therapies should not be minimized, and working on the quality and safety of such products should be increased (Barkholt et al., 2013). Several problems interfere with the clinical application of EVs from adult stem cells (SCs) in cancer treatment, such as safety issues, unpredictable pro-tumor effects, and tissue entrapment (Parfejevs et al., 2020). The risk of tumorigenesis by EVs remains a concern because of the systemic and diverse effects of their cargo. The influence of MSCs on tumor progression is subject to contradictory debate with tumor growth acting as a double-edged sword (Liang et al., 2021). Through several mechanisms and depending on many factors, MSCs may either suppress or promote tumor growth. Similar to MSCs, EVs can be either associated with tumor progression, tumorigenesis, angiogenesis, and metastasis or associated with tumor suppression, exhibiting tumor-suppressor effects (Vakhshiteh et al., 2019). In fact, the tumor microenvironment (TME) is highly affected by EVs from both tumor cells and nonmalignant cells, as they function as carriers for various molecules in the TME (Tao and Guo, 2020). Different studies have reported that MSC-EVs may exert various effects on the growth, metastasis, and drug response of different tumor cells by transferring proteins, messenger RNA, and microRNA to recipient cells (Zhang et al., 2017). Changes in the composition and secretion rate could contribute to the oncogenic effects of EVs by creating a tumor-supportive microenvironment. The cargo of MSC-derived EVs may contain factors involved in cancer metastasis and promote epithelial-mesenchymal transition (EMT). Cancer-derived EVs can thus “educate” nearby MSCs to secrete large amounts of IL-8 and other immunosuppressive cytokines. Interestingly, this inflammatory microenvironment is prone to promote the formation of new blood vessels toward the tumor (Xavier et al., 2020). Within the tumor microenvironment, stromal cells secrete EVs that will support a drug resistance phenotype in otherwise drug-sensitive cancer cells. Breast cancer cells may thus prime BM-MSCs to release exosomes containing distinct miRNA contents, such as miR-222/223, which in turn promotes quiescence in a subset of cancer cells and confers drug resistance (Bliss et al., 2016). Several studies demonstrated that treatment with MSC culture medium or MSC coculture promoted EMT in breast or gastric cancer cells (Kletukhina et al., 2019). Gastric cancer cells acquire an “activated” carcinoma-associated fibroblast (CAF) phenotype and enhance tumor metastasis and growth *in vivo* after being in close contact with MSCs. Paracrine signals induce EMT and promote transwell and transendothelial migration, and the changes are dependent on *β*-catenin, MMP-16, snail and twist (Xue et al., 2015). EVs derived from adipose tissue-derived MSCs promoted the migration and proliferation of breast cancer cells *via* the activation of the Wnt signaling pathway (Lin et al., 2013). Human umbilical cord mesenchymal stem cell-derived EVs (hUC-MSC-EVs) have been shown to significantly enhance the proliferation, migration and invasion of human breast cancer cells through the activation of the ERK pathway. hUC-MSC-EVs reduced E-cadherin expression and increased N-cadherin expression, thus promoting EMT in breast cancer cells and leading to malignant tumor progression and metastasis (Zhou et al., 2019). In addition, Zhao et al. (2018) found that the EMT-promoting effect in lung cancer was mediated by EVs secreted from hUC-MSCs through the secretion of TGF-β. MSC-EVs may promote the growth and metastasis of tumor cells by different secreted factors. Exosomes derived from BM-MSCs increase tumor growth in a BALB/c nu/nu mouse xenograft model by enhancing VEGF expression through the activation of extracellular signal regulated kinase 1/2 (ERK1/2) and the p38 MAPK pathway (Zhu et al., 2012). Bone marrow stromal cell-derived exosomes were shown to promote the proliferation, survival, and metastasis of myeloma cells by modulating the p38, p53, c-Jun N-terminal kinase, and Akt pathways (Wang et al., 2014a). Surprisingly, human Wharton’s jelly mesenchymal stem cell-derived extracellular vesicles (hWJ-MSC-EVs) were reported to promote the growth and migration of human renal cell carcinoma (RCC) by inducing HGF expression and activation of the Akt and ERK1/2 signaling pathways (Du et al., 2014), although antiproliferative and proapoptotic effects of these hWJ-MSC-EVs were described on bladder cancer cells through downregulation of Akt phosphorylation and upregulation of Caspase 3 cleavage (Wu et al., 2013). Exosomes derived from the MSCs of multiple myeloma patients expressed higher levels of oncogenic proteins, cytokines (IL-6, CCL2) and adhesion molecules (*γ*-catenin, fibronectin) and lower expression levels of the tumor suppressor miRNA-15a than exosomes derived from normal MSCs. Moreover, the latter inhibited the growth of multiple myeloma cells, whereas exosomes derived from MSCs of multiple myeloma patients promoted tumor growth (Roccaro et al., 2013). Collectively, these observations on the impact of EVs derived from MSCs on tumor biology should be well monitored and clarified to ensure the safety of the cell-free strategy.

As shown, the translation of MSC-EVs to the clinical stage is still at the initial phase. A number of concerns still have to be solved regarding their safety, particularly regarding tumors, their mechanisms of action, the possible alteration of their properties because of isolation/purification methods, and/or the best approach for large-scale clinical production (Massa et al., 2020).

#### 3.7.2 Quality Control of Mesenchymal Stem/Stromal Cells

Before their application in clinical trials or cryopreservation and throughout their production, MSCs need to undergo quality-control determination. Quality-control criteria include the determination of many characteristics, including surface markers, morphology, differentiation potential, senescence status, secretome, immunophenotype and others (Torre et al., 2015). Functional assessment before and after cryobanking is crucial, because MSCs are susceptible to alteration in their function and characteristics under freeze-thawing conditions. Furthermore, at the level of clinical grade production, culturing and manufacturing conditions are able to highly influence MSCs; thus, they must be in compliance with the principles of good manufacturing practices (GMPs) to ensure their safety and efficacy (Sensebe et al., 2013). Several techniques and assays had to be performed to assess safety and efficacy; tests for contamination, including endotoxin assays, sterility tests and Gram staining, and detection of *mycoplasma*, had to be performed to ensure safe production (Galvez et al., 2014). In addition, genome stability is pivotal to prevent oncogenic risks and should be assessed by performing tests such as comparative genome hybridization (CGH) or fluorescence *in situ* hybridization (FISH) (Sensebe et al., 2013). Finally, each study, according to its needs and goals, had to perform several tests and assays and had to follow specific rules to benefit from a final product with high quality and potential.

#### 3.7.3 Clinical Prediction Tools Including OMICS

Far away from the traditional techniques that measure cell surface markers and cellular morphology, omics-based biomarkers such as proteomics, genomics, epigenomics, metabolomics and transcriptomics are new revolutionary methods for distinguishing MSCs with different features that may lead to the failure or success of the treatment at a clinical level. In other words, the characterization of omics in different culture conditions (monolayer cell culture versus aggregate cell culture) explained the therapeutic potential of MSCs and suggested that some of the failed clinical trials were due to the different abilities of MSCs in monolayer cultures versus *in vivo*. The results demonstrated that the aggregate culture enhanced the secretory capacity of MSCs and altered the metabolism of several proteins and lipids (Doron et al., 2020). Furthermore, transcriptome analysis is key for understanding the functional and differentiation potency of MSCs. Studies have shown that transcriptional profiling could be a predictive tool for stem cells (Wells and Choi, 2019). Clinical prediction tools assist in clinical outcome prediction, and omics approaches have recently served in many studies to identify the targets of several treatments based on MSCs.

## 4 Conclusion

MSCs have been investigated as a therapeutic strategy for several medical indications. The fate and behavior of MSCs are regulated by their environment, which may consequently influence their repair potential. The mechanisms of action of MSCs are mainly linked to their secretome, including chemokines, cytokines, growth factors and nucleic acids. These regulatory elements can be secreted separately or packaged into extracellular vesicles. As MSCs are able to sense and respond appropriately to local tissue challenges, such plasticity raises the possibility of preconditioning (licensing or priming) MSCs to adopt a distinct fate and function while targeting specific diseases. Currently, applied MSCs should be handled with precaution, as minor unknown or less characterized effects may hamper their therapeutic effect. Discussing new insights into the biological properties of MSCs, as well as the different preclinical and clinical challenges, will help to develop and optimize a safe and efficient therapeutic strategy.

## Glossary

MSCs: Mesenchymal stem/stromal cells
ISCT: International Society for Cellular Therapy
IFN: Interferon
TNF: Tumor Necrosis Factor
IDO: Indoleamine 2,3-dioxygenase
GvHD: Graft-versus-host disease
CVD: Cardiovascular disease
VEGF: Vascular endothelial growth factor
HGF: Hepatocyte growth factor
IL: Interleukin
CXCR4: C-X-C Motif Chemokine Receptor 4
RA: Rheumatoid arthritis
MSC-EVs: MSC-Extracellular vesicles
EVs: Extracellular vesicles
BM-MSC: Bone Marrow derived MSCs
MSC-Exos: MSC-derived exosomes
Exos: Exosomes
MPs: Microparticles
hUC-MSCs: Human umbilical cord-derived MSCs
AT-MSCs: Adipose tissue-derived MSCs
MSC-NTF: Neurotrophic factor-secreting MSC
ALS: Amyotrophic Lateral Sclerosis
IV: Intravenously
DMARDs: disease-modified antirheumatic drugs
FDA: Food and Drug Administration
CD: Crohn disease
CFU-F: Colony-forming unit-fibroblasts
NG2: Neuron-glial antigen 2
IGF-1: Insulin-like growth factor 1
EPO: Erythropoietin
GDNF: Glial Cell Derived Neurotrophic Factor
MMPs: Matrix Metalloproteinases
FGF-2: Fibroblast growth factor-2
IGFBP-6: Insulin-like growth factor-binding protein 6
MHC-I: Major histocompatibility complex-class1
HLA: Human leukocyte antigen
FAS: Fas Cell Surface Death Receptor
GM-CSF: Granulocyte-macrophage colony-stimulating factor
MIF: Macrophage migration inhibitory factor
bFGF: Basic fibroblast growth factor
PGE2: Prostaglandin E2
COX-2: Cyclooxygénase-2
OA: Osteoarthritis
RCTs: Randomized clinical trials
TLRs: Toll-like receptors
RANTES: Regulated upon Activation, Normal T Cell Expressed and Presumably Secreted
ICAM: InterCellular Adhesion Molecule
VCAM-1: Vascular cell adhesion molecule
IBMIR: Instant-blood mediated inflammatory reaction
TF: Tissue factor
DSCs: Decidua stromal cells
mCRPs: Membrane complement regulatory proteins
HSA: Human serum albumin
ECM: Extracellular matrix
MSCs-CM: MSCs-conditioned medium
TME: Tumor microenvironment
EMT: Epithelial-mesenchymal transition
hWJ-MSC-EVs: Human Wharton’s jelly mesenchymal stem cells derived extracellular vesicles
